# Influence of the Water Model on the Structure and Interactions of the GPR40 Protein with the Lipid Membrane and the Solvent: Rigid versus Flexible Water Models

**DOI:** 10.1021/acs.jctc.4c00571

**Published:** 2024-07-11

**Authors:** Jorge Alberto Aguilar-Pineda, Minerva González-Melchor

**Affiliations:** Instituto de Física “Luis Rivera Terrazas”, Benemérita Universidad Autónoma de Puebla, Av San Claudio, Cd Universitaria, Apdo. Postal J-48, Puebla 72570, México

## Abstract

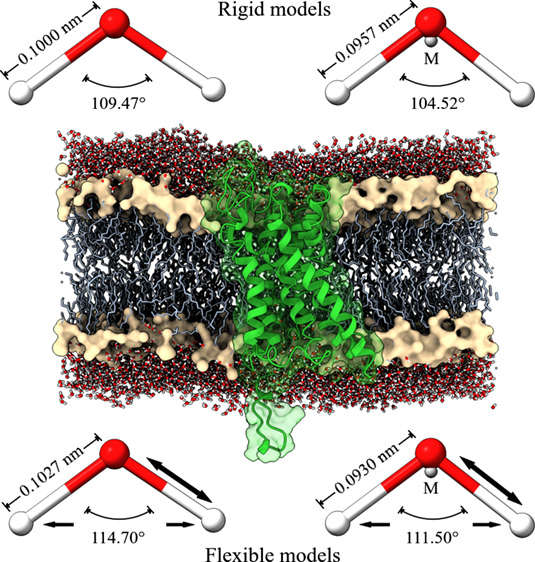

G protein-coupled receptors (GPCR) are responsible for modulating various physiological functions and are thus related to the pathophysiology of different diseases. Being potential therapeutic targets, multiple computational methodologies have been developed to analyze their behavior and interactions with other species. The solvent, on the other hand, has received much less attention. In this work, we analyzed the effect of four explicit water models on the structure and interactions of the GPR40 receptor in its apo form. We employed the rigid SPC/E and TIP4P models, and their flexible versions, the FBA/ϵ and TIP4P/ϵ_flex_. We explored the structural changes and their correlation with some bulk dynamic properties of water. Our results showed an adverse effect on the conservation of the secondary structure of the receptor with all the models due to the breaking of the intramolecular hydrogen bond network, being more evident for the TIP4P models. Notably, all four models brought the receptor to states similar to the active one, modifying the intracellular part of the TM5 and TM6 domains in a “hinge” type movement, allowing the opening of the structure. Regarding the dynamic properties, the rigid models showed results comparable to those obtained in other studies on membrane systems. However, flexible models exhibit disparities in the molecular representation of systems. Surprisingly, the FBA/ϵ model improves the molecular picture of several properties, even though their agreement with bulk diffusion is poorer. These findings reinforce our idea that exploring other water models or improving the current ones, to better represent the membrane interface, can lead to a positive impact on the description of the signal transduction mechanisms and the search of new drugs by targeting these receptors.

## Introduction

The microenvironment surrounding cell-membrane proteins is very complex due to the variety of components that constitute it.^1,2^ However, it is known that water, its main component, plays a vital role in the biochemical maintenance of living cells.^3^ Physiological processes like structural stability, self-assembly ability, protein interaction, and molecular recognition are mainly due to hydration forces, hydrophobic effects, and the complex network of hydrogen bonds between water molecules and protein residues.^4−6^ Furthermore, the affinity of protein–ligand complexes is related to the ability of ligands to displace water molecules from an active site.^7^ Despite the advances made to understand the key role of water in maintaining life, its action as a driving force of biological processes continues to be a significant challenge in research in various fields of experimental, theoretical, and computational science.

At this point, numerous studies have focused on protein–water systems, with soluble proteins being the primary focus.^4,8^ Nevertheless, the environment surrounding membrane proteins differs from that of plasma proteins. Experimental evidence suggests that properties such as density, diffusion rates, and dielectric constants can decrease in these systems due to the reduction in the mobility of water molecules, impacting the biological function of the protein.^9−11^ Despite this, significant efforts have been made to understand the role of water in protein–membrane systems, both as a solvent with its bulk properties and in forming the hydration shell.^12−14^

Several transmembrane proteins have been studied for these purposes, but a system that has generated the greatest interest in research groups worldwide is the family of G protein-coupled receptors (GPCRs). These receptors are involved in a wide range of physiological processes,^15,16^ and therefore, any damage or malfunction has been associated with various types of diseases or their pathogenesis.^16,17^ Constituted by seven transmembrane helices connected by three extracellular loops and three in the cytoplasmic region, these receptors represent almost 4% of the proteins expressed by the human genome.^18^ In this context, one of the diseases related to the malfunction of the activity of GPCRs is diabetes, especially type 2 diabetes.^19^ Globally, it is estimated that around 537 million people are affected by diabetes, projected to rise to 783 million by 2045.^20^ According to Hauser et al., roughly 9% of drugs in clinical trials target GPCRs associated with diabetes and obesity.^16^ Diabetes is a metabolic disorder characterized by abnormally high blood glucose levels resulting from inadequate insulin secretion or insulin resistance by cells. Various GPCRs have been linked to the insulin release stimulation mechanism, among which the GPR40 receptor stands out, which belongs to the family of free fatty acid receptors (FFARs).^21^

Computationally, the GPR40 receptor has been widely studied in designing new drugs,^21−25^ understanding activation/deactivation mechanisms,^26,27^ and searching for allosteric sites.^28−30^ Several in-silico techniques have been used for these purposes, with molecular dynamics (MD) being the most used technique. One of the advantages of this receptor is that there are experimental structures, both in active states (8eit, 8ejc, and 8ejk^31^) and inactive states (4phu,^32^ 5tzr, 5tzy,^33^ and 5kw2^34^) that can be used as initial configurations. Furthermore, databases such as UniProt^35^ or RCSB PDB^36^ have incorporated into their repositories structures modeled by the AlphaFold program, which makes three-dimensional predictions of proteins based on artificial intelligence (AI).^37,38^ In the case of GPR40 (code AF–O14842-F1), the modeled structure shows a complete three-dimensional sequence, making it ideal for in-silico studies.^39^

However, having a good structural representation of the protein system to be studied does not ensure reliable results. Issues such as the water model used in the molecular simulations are not correctly raised, leaving its function only to solvate the system. The choice of solvent is often made based on models that ensure high performance at low computational costs. Despite new water models that present improvements to specific systems, popular models such as SPC,^40^ SPC/E,^41^ TIP3P,^42^ or TIP4P^43^ continue to be preferred. These models are also widely used in studies with GPCR receptors, including GPR40.^22−26^ Although it is difficult to evaluate the suitability of a model for certain systems, the introduction of flexibility to rigid models has improved our molecular image of them.^44^ In this sense, two flexible water models based on the original SPC/E and TIP4P/ϵ models, namely, the FBA/ϵ and TIP4P/ϵ_flex_ models^45,46^ are now available. These models, developed to reproduce the experimental density, dielectric constant, and infrared spectrum, add harmonic potentials to the O–H bonds and the H–O–H angle. Adding flexibility to represent water molecules is relevant when intramolecular fluctuations are essential to improve properties such as diffusion and surface tension, in addition to those already mentioned.^47^

In this study, we explored the effect of two flexible water models on the structural and energetic properties of the GPR40 receptor: the three-site FBA/ϵ model and the four-site TIP4P/ϵ_flex_ model. The results were contrasted with their rigid counterparts, SPC/E and TIP4P. Using molecular dynamics simulations and different in-silico techniques, our results show that all four models fail to preserve the secondary structure of the receptor. Notably, all four models bring the receptor into an open configuration, characteristic of active configurations. Although the flexible models have a more significant interaction via hydrogen bonds with the receptor, the FBA/ϵ model is the one that preserved the GPR40 structure to a greater extent. Furthermore, this model showed that several of the properties evaluated are comparable, and some even improve, to the results obtained with the rigid models. On the other hand, the TIP4P/ϵ_flex_ model was the one that showed the lowest performance in the evaluated properties. Moreover, based on our results, we have tried to explore the conformational changes of the GPR40 receptor and the impact of water molecules on properties such as diffusion, density, and dielectric constant. The rest of the manuscript is organized as follows. In the Computational Details Section, we present the details and preparation of the GPR40-membrane-water systems. In the Results and Discussion Section, we develop the findings and discussions of the present work. First, we present an analysis of the initial structure of GPR40. Second, we analyzed the stability descriptors of the four water models. Then, we present a structural comparison with other GPCR structures in inactive and active states. Finally, we attempted to correlate some water properties with configurational changes in GPR40-membrane systems. Lastly, the main conclusions and references are given.

## Computational Details

### Structure Models

In order to assess the influence of the water model on the structural conformations and interactions of protein–membrane systems, four nonpolarizable water models were considered, namely, SPC/E,^41^ TIP4P,^42^ FBA/ϵ,^45^ and TIP4P/ϵ_flex_.^46^ The first two models have been widely used in the study of biomolecules in aqueous solutions and are characterized by being rigid models of three and four sites, respectively. The last two are reparameterized and improved models, incorporating flexibility in the O–H bond and the HOH angle. Table 1 compares the force field parameters of these models.

**Table 1:** Force Field Parameters of Non-Polarizable Water Models Used in This Work

model	*r*_OH_ (nm)	*k*_b_(kJ mol^–1^ nm^–2^)	θ (°)	*k*_a_ (kJ mol^–1^ rad^–2^)	ϵ_OO_ (kJ mol^–1^)	σ_OO_ (nm)	*q*_H_ (e)
SPC/E	0.10000		109.47		0.650155	0.31660	0.4238
FBA/ϵ	0.10270	300,000	114.70	383.00	0.792324	0.31776	0.4225
TIP4P	0.09572		104.52		0.636386	0.31540	0.5200
TIP4P/ϵ_flex_	0.09300	157,000	111.50	212.00	0.794032	0.31734	0.5100

Due to the high incidence of diabetes cases in our population, of particular interest to our research group is the protein structure of the GPR40 receptor, also known as FFAR1. This receptor has been related to the pathogenesis of type II diabetes. Atomic coordinates were retrieved from the AlphaFold server database^38,48^ with the identifier AF–O14842-F1.^39^

In all systems, the dipalmitoyl-phosphatidyl-choline (DPPC) lipid bilayers were used as a membrane model. This lipid was chosen since phosphatidylcholine is the main constituent in mammalian cell membranes (40 mol % of lipid mass) and a precursor of more complex membranes.^49^ Furthermore, the DPPC model is widely used experimentally in studies of drugs targeting cells located in the pancreatic islets in the treatment of diabetes.^50−52^

### Water as a Pure Component

In order to prepare the GPR40-membrane-water systems, previous MD simulations of the four water models as a pure component were performed to ensure their reproducibility. These systems were run under the same conditions as the production simulations of the biomolecular systems, i.e., in an NPT ensemble at 309.65 K and one bar of pressure by 100 ns. The four systems consisted of 500 molecules.

### Preparation of Molecular Systems

In all systems, the GPR40-membrane complexes were built following the methodology proposed by Lemkul.^53^ First, the lipid membrane was constructed from a bilayer model of 128 DPPC molecules which was replicated twice in the *x* and *y* directions. Once the membrane with 512 lipid molecules was obtained, the structure of the receptor was embedded following the InflateGRO methodology,^54^ updating the number of lipids per bilayer (509 molecules). From this, the residues embedded in the lipid matrix were determined using the DeepTMHMM server.^55^ To avoid unwanted effects originated by the use of periodic boundary conditions, a gap of 1.8 nm was left between the limits of the simulation cell on the *z*-axis and the molecular surface of the protein. The dimensions in the *x* and *y* axes were set according to the dimensions of the lipid membrane (∼12.9 nm). Then, four replicas of the GPR40-membrane complex were made, and each system was solvated with one of the proposed water models. For this purpose, the *gmx solvate* tool was used. In all systems, the water molecules located in the hydrophobic region, defined between the aliphatic chain and the ester group of the lipids of both layers, were eliminated using a homemade program.^53^ Finally, chlorine ions were added to neutralize the total charge of the simulation cells. Next, sodium and chlorine ions were added at a concentration of 0.154 M to mimic physiological conditions.

### MD Simulations

All molecular dynamics simulations were performed using the Gromacs 2021 package^56,57^ and the OPLS-AA force field parameters.^58,59^ The interaction parameters used for the membrane were those obtained by Tieleman and Berendsen.^60^ To achieve optimal results, the first energy minimization was performed using the *Steepest Descent* algorithm for 50,000 steps, considering only the membrane structures and the GPR40 protein in a vacuum. Afterward, the systems were solvated with the different water models, and 0.154 M NaCl was added to neutralize them electrically. Once the complete systems were obtained, a second energy minimization was performed, followed by two equilibrium simulations in the NVT (50 ps) and NPT ensembles with position restraints in heavy atoms and a temperature of 323.15 K. The NVT simulation was carried out using the V-rescale thermostat^61^ while for the NPT simulation, the Nosé–Hoover thermostat^62,63^ and the Parrinello–Rahman barostat with a semi-isotropic pressure coupling were used.^64^ The compressibility factor was 4.5 × 10^–5^ 1/bar. The NPT equilibrium simulation trajectory was 3 ns, saving positions and velocities every nanosecond. These frames served as initial structures in the production simulations of each replica.

The MD production trajectories were performed without position restraints in the NPT ensemble at 309.65 K with semi-isotropic pressure coupling for 500 ns. The equations of motion were integrated using a leapfrog integrator with a time step of 1 *f*s. Periodic boundary conditions (PBCs) in *x*, *y*, and *z* directions were used. A particle mesh Ewald (PME)^65^ algorithm for long-range electrostatics with cubic interpolation, a cutoff of 1.2 nm, and a linear constraint solver (LINCS) with all bonds constrained were applied for all MD simulations.^66,67^ A Nosé–Hoover thermostat with a coupling time of τ_T_ = 0.5 ps and the Parrinello–Rahman barostat with a semi-isotropic pressure coupling and a relaxation time constant of τ_P_ = 2.0 ps were used. All production trajectories were saved every 10 ps.

### Structure and Data Analysis

The statistical results, root-mean-square deviation (RMSD), mean square displacement (MSD), root-mean-square fluctuation (RMSF), radii of gyration (RG), solvent-accessible surface area (SASA), structures, trajectories, and B-factor maps, were obtained using the Gromacs modules. Hydrogen bonds (HB) were calculated using the geometric criteria predetermined by Gromacs,^68^ i.e., *r* ≤ *r*_HB_ = 0.35 nm and α < α_HB_ = 30°. These same parameters were used to calculate the percentage of HB occupancy along the production trajectories using the VMD software and its *Hydrogen Bonds* analysis tool. Analysis of the structure properties was performed using the production MD trajectories of the last 200 ns of each simulation and then visualized using the Visual Molecular Dynamics (VMD)^69^ and the UCSF Chimera v.1.14^70^ software. Graphs were plotted using the XMGrace software.^71^ Electrostatic potential (ESP) surfaces in the molecular mechanics framework were calculated with the APBS software v.1.4.1,^72^ and the PQR input was created in the PDB2PQR^73^ server. Free energy landscape (FEL) maps were used to visualize the energy associated with protein conformation of the different models during MD simulations. These maps were plotted using the *gmx sham* module while the RMSD and RG were calculated from the atomic position variables with respect to their mean structure and from the center of mass of the protein, respectively. Figures related to the FEL were constructed using Wolfram Mathematica 12.1.^74^ Stability descriptors, properties, and standard deviation fluctuations reported in this work are the average values obtained from the data of the three replicates.

## Results and Discussion

This section first discusses the relevant aspects of constructing the studied systems. Afterward, the influence of water models on the structural properties of the GPR40 receptor using stability descriptors to address the configurational changes will be analyzed. Finally, some dynamic properties of water will be addressed to correlate them to the behavior of the receptor under the solvent environment. In order to ensure the reproducibility of our results and assess their statistical significance, three parallel MD production simulations were performed for each system with trajectories of 500 ns each. The three replicas of each system showed similar trends. For this reason, the average values of the properties and descriptors that have been calculated are discussed throughout the manuscript. The GPR40 receptor structures used to illustrate the discussion correspond to one of the replicas. Additional information on the other two replicas is provided in the Supporting Information.

In order to facilitate the reading and understanding of the following results, different colors and abbreviations were used to refer to each water model. In this way, both the structure of the GPR40 and the statistical data were colored green when the SPC/E water model was used. Blue, orange, and purple colors were used for the TIP4P, FAB/ϵ, and TIP4P/ϵ_flex_ models, respectively. In this same order, the abbreviations G40-spce, G40-t4, G40-fba, and G40-t4f were used when referring to the GPR40 structure and the whole system.

### Initial Structure of GPR40

**Figure 1:**
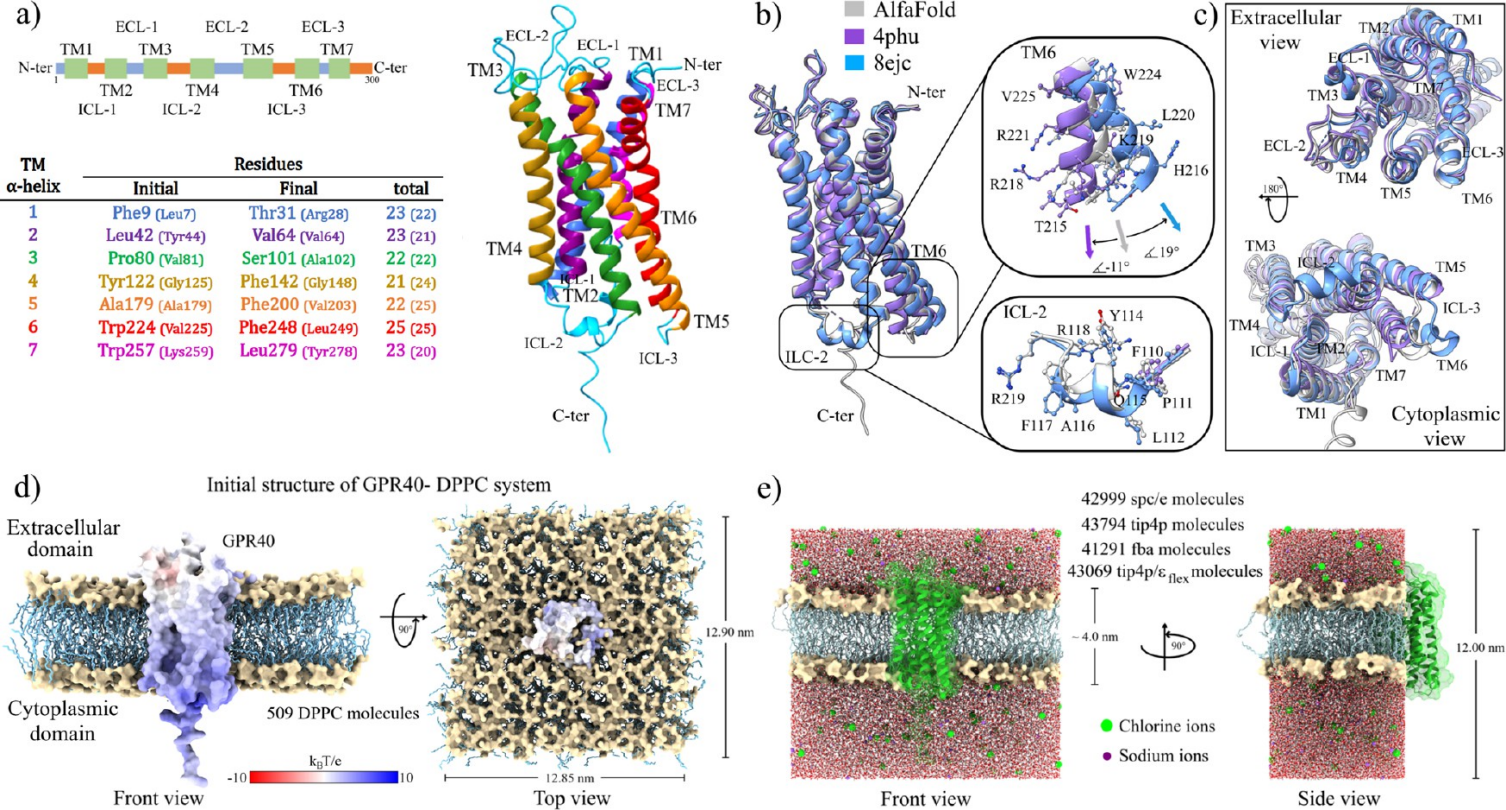
(a) Topology and building of the GPR40-membrane-water systems. Main domains of the GPR40 receptor. The table shows the residues that comprise the structure according to the work of Srivastava et al. and, in parentheses, those obtained by the DeepTMHMM server. Different colors were used to represent each transmembrane domain on the table and its 3-D representation for easy visualization. (b) Structural superimposition of the AphaFold (gray color), 4phu (inactive state, purple color), and 8ejc (active state, blue color) models. The insets show the ICL-2 and TM6 domains, which are associated with the activation states of GPRC receptors. (c) Views of the intracellular and cytoplasmic regions of the structural alignment. (d) Schematic representation of the embedded GPR40 receptor in the DPPC lipid membrane. (e) Size of the simulation cell in the *z*-direction was set so there were no problems due to periodic boundary conditions between the protein atoms. Depending on the water model, different numbers of molecules were needed to solvate the systems.

Expressed by the FFR1 gene, the structure of GPR40 consists of 300 residues, 159 of which are part of the seven transmembrane (TM) α-helices characteristic of GPCR proteins.^32^ The N-terminal domain (M1-S8) and the three loops called ECL (extracellular loop, E65-C79, G143-S178, and L249-S256) are in the extracellular region. On the other side, the three domains called ICL (intracellular loops, A32-S41, A102-C121, and C201-A223) and the C-terminal domain (G280-K300) are located in the cytoplasmic region. This last region comprises an α-helix between residues G284 and Q294, the rest being the loop type (Figure 1a).

To build the initial molecular systems, the AlphaFold model of the GPR40 (AFG40) receptor was chosen. This structure solves the problem of nonlocalized sections of the experimental models and gives a nonmutation model. Furthermore, the modeling of this structure seems to have considered the configurations of the inactive and active states of the receptor adopting an intermediate active shape. This can be appreciated by performing a structural superimposition with the 4phu (inactive state) and 8ejc (active state) models, where the RMSD values were 0.99 and 1.51 respectively, regarding the AFG40 structure (Figure 1b). The main structural differences between these models were the ICL-2 and TM6 domains (insets in Figure 1b). Notably, the positions of the TM6 domain, concertedly with a movement of TM5, have been related to active or inactive states of GPCR structures.^15^ In other words, an opened configuration of this domain indicates an active-like state and, a closed shape, an inactive-like state. In this sense, the AFG40 shows an in-between angle regarding the 4phu (∼11°) and the 8ejc structures (∼ −19°), suggesting that the intracellular region of the TM6 domain is in an intermediate active conformation. On the other hand, the ICL-2 domain is only present in the AFG40 and 8ejc models, while it was not detected in the 4phu structure, possibly because it acquires a disordered conformation. The importance of this domain lies in that it is considered a key in the coupling of the GPCRs and downstream effectors, like G-proteins.^33,34,75^

This intermediate configuration is also observed in the rest of the transmembrane helices. Figure 1c shows that the domains in the extracellular region TM2, TM3, TM4, and TM5 of the 4phu structure are in an “inward” configuration concerning the 8ejc model. This same “inward” configuration is observed with all TM domains on the cytoplasmic side, and in both cases, the AFG40 configuration is found between these two structures.

The topology of the different domains of the AFG40 structure was determined using the DeepTMHMM server (Figure S1a in the Supporting Information).^55^ The results obtained and the structural data provided by Srivastava et al. made it possible to establish the regions immersed in the lipid membrane of the TM domains (Table of Figure 1a).^32^ An additional criterion in this last step was to consider the hydrophobic match between the TM domains and the lipid bilayer.^76^ For this, an analysis of the electrostatic potential surface of the AFG40 model and the TM regions predicted by the DeepTMHMM server was performed. Results showed two areas with different electrostatic properties (Figure S1b). On the extracellular side, in both of the ECL and TM domains, the surface shows a hydrophobic character (white color), with a small nucleophilic pocket located between residues V141, L144-P147, L151-D152, D175-S178, and P181-A182 of the TM4 and TM5 domains (red color). However, on the cytoplasmic side, regions with electrophilic characteristics are predominant in the structure, increasing toward the core of the protein. This positive charge property on the cytoplasmic region has been noted in other GPCR structures.^77^ Kang et al. suggest that the electrophilic character in GPCRs could serve in complex formation with G-proteins or arrestins through charge complementarity mechanisms.^78^ Regarding the TM domains, the maximum thickness of the region embedded in the lipid matrix was 4.08 nm, the distance between residues W124 and P147 of the TM4 domain (Figure S1c).

Figure 1d shows the embedding of the GPR40 receptor in the center of the lipid bilayer. In the solvation process, the dimensions of the simulation cell were kept constant, so the number of water molecules varied in each system (Figure 1e). Prior to the production simulations and in order to validate the coupling in the GPR40-membrane-solvent systems, the thickness and area per lipid parameters of the membrane were calculated at the end of the equilibrium simulations. The results showed an average lipid bilayer thickness of 3.98 nm, with an area per lipid of 60.5 Å^2^, which is very close to those obtained experimentally at the same temperature of 323.15 K (∼3.80 to 3.83 nm^79,80^ and 63.1 Å^2^,^81^ respectively).

### Flexibility of the C-terminal Domain Increases the Energy Landscape of Apo-GPR40 Structures

**Figure 2:**
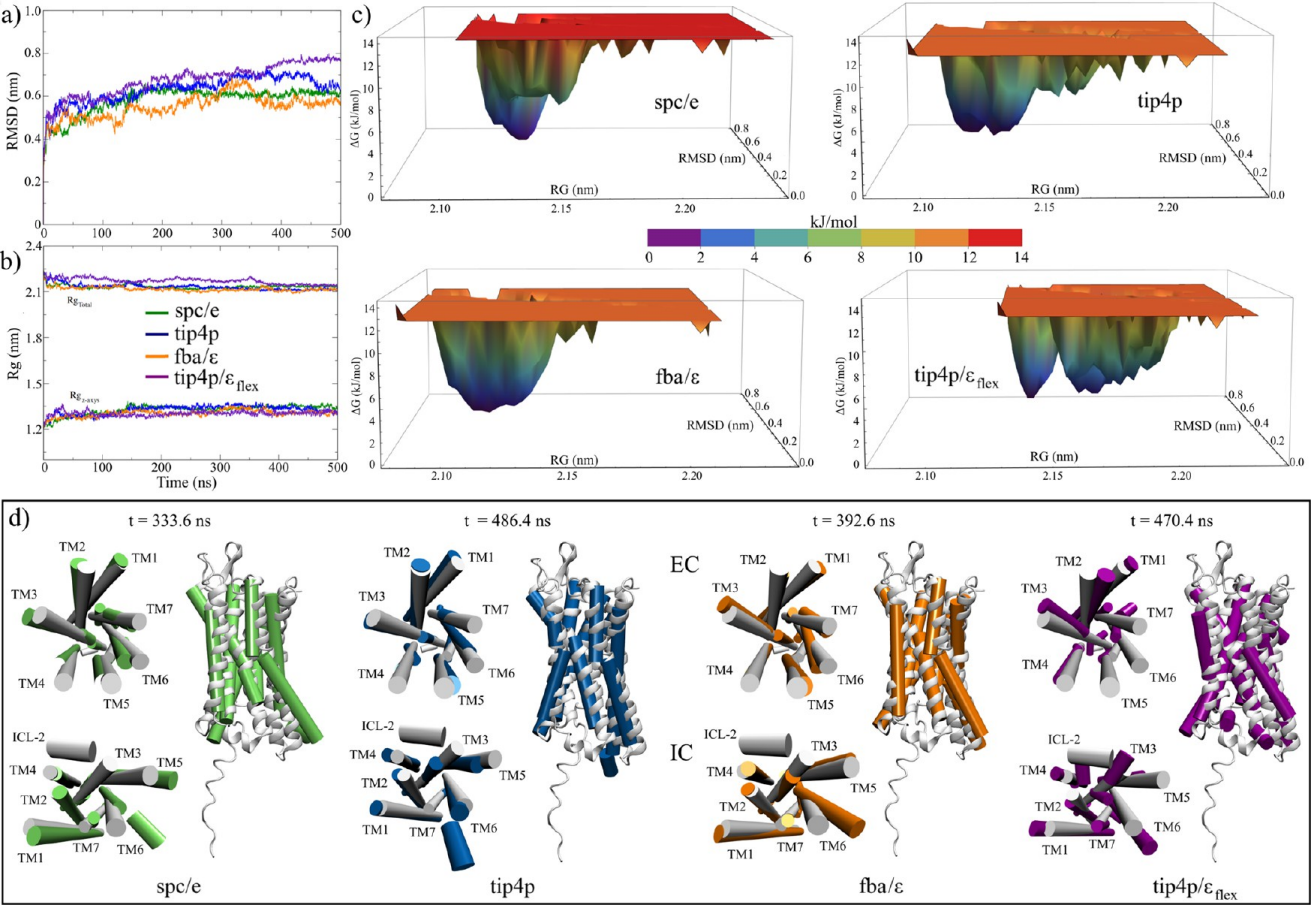
Analysis of the MD trajectories of the complete structure of the GPR40 receptor in its apo form (300 residues). (a) RMSD graph of the four systems studied. (b) Total radius of gyration and on the *z*-axis, perpendicular to the plane of the membrane. Line colors indicate the water model used in the systems. (c) Heat maps of the FEL analysis. The free energies were obtained, taking the RMSD values and the radius of gyration as variables. The axes show the same scales of the three calculated variables for a better comparison. (d) Minimum energy structures found in FEL analysis.

In order to evaluate the ability of each water model to stabilize the GPR40 structure within the lipid membrane, an initial analysis using the average values of stability indicators outlined in Table 2 was conducted. Plots of these descriptors for each simulated replica can be consulted in Figures S2 and S3 of the Supporting Information. As a first indicator, the root-mean-square deviation (RMSD) was analyzed, a parameter widely used to analyze the conformational changes and dynamic equilibrium in MD trajectories. In the first evaluation, the results obtained from our calculations were not as expected. Previous in-silico studies have shown that the structure of the apo-GPR40 form was stable under the conditions imposed in the MD simulations and did not vary significantly from its crystalline conformation.^23,24,27,30^ Using the SPC and TIP3P three-point water models, the authors reported RMSD values ranging from 0.15^24^ to 0.35 nm,^27^ revealing a great conformational equilibrium. Nevertheless, although our systems reached a certain level of stability after the first 150 ns, all values of this parameter were over 0.59 nm in the last 200 ns of the MD trajectories (Figure 2a). These results are comparable to those obtained in other studies where GPR40 was not embedded in a lipid bilayer.^22,25^ Furthermore, it can be seen that flexible models exhibit greater fluctuation in RMSD values compared to their rigid counterparts.

**Table 2:** Stability Descriptors of the Studied Systems

water model	RMSD^a^	radius of gyration^a^	RMSF^a^	SASA^b^	H-bonds^c^			
					intra	prot-solv	prot-mem	mem-solv
SPC/E	0.61 ± 0.01	2.13 ± 0.01	0.14 ± 0.07	160.39 ± 1.45	189 ± 4	286 ± 6	37 ± 3	1551 ± 16
	*0.39 ± 0.01*	*2.05 ± 0.01*	*0.13 ± 0.06*	*149.76 ± 1.26*				
FBA/ϵ	0.59 ± 0.05	2.11 ± 0.01	0.16 ± 0.13	158.42 ± 1.40	187 ± 4	304 ± 6	33 ± 3	1660 ± 17
	*0.32 ± 0.02*	*2.02 ± 0.01*	*0.12 ± 0.06*	*147.36 ± 1.43*				
difference^d^	+0.02	+0.02	–0.02	+1.97	+2	–18	+4	–109
	*+0.07*	*+0.03*	*+0.01*	*+2.4*				
TIP4P	0.68 ± 0.02	2.12 ± 0.01	0.15 ± 0.09	161.31 ± 1.39	184 ± 5	301 ± 7	38 ± 3	1727 ± 17
	*0.33 ± 0.01*	*2.03 ± 0.01*	*0.12 ± 0.06*	*148.98 ± 1.47*				
TIP4P/ϵ_flex_	0.74 ± 0.03	2.16 ± 0.02	0.18 ± 0.11	165.29 ± 1.71	162 ± 4	348 ± 7	37 ± 3	1707 ± 17
	*0.38 ± 0.02*	*2.09 ± 0.01*	*0.15 ± 0.07*	*157.76 ± 1.19*				
difference^d^	–0.06	–0.04	–0.03	–3.98	+22	–47	+1	+20
	*–0.05*	*–0.06*	*–0.03*	*–8.78*				

a In nanometers.

b In square nanometers.

c Number of H-bonds formed. The italicized were obtained without taking into account the C-terminal domain (second row for each model).

d Values calculated by *V*_rig_ – *V*_flex_. All values were obtained from the last 200 ns of the MD trajectories.

However, the difference between our results for this descriptor and those mentioned above is the inclusion of the C-terminal group (G280-K300). We used the whole receptor structure in the RMSD calculation, and the aforementioned studies used models that do not include this domain (4phu,^23^ 5tzr,^24,30^ and 5tzy^27,30^). Although doubts remain about the function of this domain, Srivastava et al. suggest that it could serve as a peptide-binding region or to distinguish between activated or deactivated forms.^32^ Thus, when this domain is excluded, the RMSD values obtained are consistent with the aforementioned studies. Noteworthy, the average values of this descriptor converge in all the systems studied if this domain is not considered (Figure S4a).

Similarly, it was observed that the inclusion or exclusion of the C-terminal domain affects the average values of the radius of gyration (Rg, Figures 2b and S4a). Furthermore, considering that the initial value of the total Rg was 2.21 nm (AlphaFold structure, AFG40), the decrease in this descriptor would indicate the compaction of the GPR40 structures (Table 2). Although this behavior is expected by folding the C-terminal domain, which is initially perpendicular to the membrane, it does not determine the displacement of the TM domains and, therefore, their compaction. Thus, this descriptor was recalculated without taking into account the C-terminal domain. The results showed that the receptor acquires “open” configurations concerning the TM domains (italics values in Table 2) compared to the new initial value of 1.99 nm. In order to clarify these findings, the Rg analysis was performed by components (Rg_*x*_, Rg_*y*_, Rg_*z*_), excluding the C-terminal domain. Values in Table 3 (columns 7–10) show no significant changes in the compaction on the *x* and *y* axes regarding the reference structure (AF-noTail). A slight increase concerning the *y*-axis was observed in the G40-t4f structure (5.6% of the initial value). However, notable results were found when analyzing the Rg_*z*_ descriptor. The “open” conformation of the receptor is due to the aperture of the TM domains, which is observed in the increase in Rg_*z*_ values, reaching a maximum value in the G40-t4f structure (10.9%).

**Table 3:** Comparison of Structural Parameters of the Simulated Models with Crystalline Structures Obtained Experimentally, Both Inactive and Active State Structures

GPCR	resnum	secondary^a^ structure summary (%)				radius^b^ of gyration				volume/area	RMSD^c^ (Å)
		β-strand	α-helix	3–10 α-helix	other	total	*x*_axis_	*y*_axis_	*z*_axis_		
AlphaFold	300	9 (3.0)	211 (70.3)	3 (1.0)	77 (25.7)	2.21	2.00	2.06	1.21	35.24/14.30	
AF-noTail	300	9 (3.0)	211 (70.3)	2 (0.7)	78 (26.0)	1.99	1.82	1.79	1.19	34.97/13.40	0.55
SPC/E	300	4 (1.3)	160 (53.3)	0 (0.0)	136 (45.4)	2.05	1.83	1.82	1.31	35.62/14.59	6.88
TIP4P	300	4 (1.3)	162 (54.0)	12 (4.0)	119 (39.7)	2.03	1.80	1.81	1.31	35.49/15.04	5.91
FBA/ϵ	300	0 (0.0)	154 (51.3)	22 (7.3)	124 (41.4)	2.02	1.81	1.80	1.30	35.48/14.65	4.15
TIP4P/ϵ_flex_	300	9 (3.0)	120 (40.0)	12 (4.0)	159 (53.0)	2.09	1.86	1.89	1.32	36.41/15.89	8.40
inactive state structures											
7epf	240	0 (0.0)	190 (79.2)	5 (2.1)	45 (18.8)	1.85	1.61	1.70	1.16	29.25/10.98	17.95
5kw2^d^	247	0 (0.0)	200 (81.0)	4 (1.6)	43 (17.4)	1.86	1.66	1.71	1.14	29.45/11.23	1.70
4phu^d^	270	4 (1.5)	204 (75.6)	0 (0.0)	62 (23.0)	1.94	1.75	1.76	1.16	31.30/11.94	0.99
5tzr^d^	273	4 (1.5)	203 (74.4)	2 (0.7)	64 (23.4)	1.94	1.74	1.77	1.16	31.63/12.11	0.97
5tzy^d^	274	4 (1.5)	206 (75.2)	2 (0.7)	62 (22.6)	1.95	1.77	1.78	1.16	31.67/12.42	1.20
3vw7^d^	282	8 (2.8)	208 (73.8)	2 (0.7)	64 (22.7)	2.01	1.79	1.85	1.19	35.12/13.12	4.63
active-state structures											
8ejk^e^	267	0 (0.0)	206 (77.2)	2 (0.7)	59 (22.1)	1.98	1.77	1.80	1.25	31.21/12.84	1.50
8eit^e^	268	4 (1.5)	198 (73.9)	2 (0.7)	64 (23.9)	1.97	1.77	1.79	1.24	31.26/13.46	1.69
8ejc^e^	271	0 (0.0)	210 (77.5)	2 (0.7)	59 (21.8)	1.98	1.78	1.79	1.24	32.10/12.26	1.52
5nx2	282	0 (0.0)	223 (79.1)	6 (2.1)	53 (18.8)	2.12	1.89	1.92	1.32	36.09/15.59	6.24
2rh1	282	0 (0.0)	217 (77.0)	3 (1.1)	62 (22.0)	2.04	1.78	1.88	1.27	35.98/13.83	5.08
5g53	283	4 (1.4)	223 (78.8)	4 (1.4)	52 (18.4)	2.08	1.90	1.78	1.36	35.06/13.77	6.68
3sn6	284	0 (0.0)	225 (79.2)	3 (1.1)	56 (19.7)	2.09	1.85	1.89	1.31	33.92/14.60	5.15
4zwj	326	10 (3.1)	224 (68.7)	0 (0.0)	92 (28.2)	2.19	1.94	2.03	1.29	41.60/15.52	6.65

a The values indicate the total number of residues that comprise the secondary structure, while the values in parentheses represent the percentages concerning the total residues of the protein structure.

b The values of the initial structures and those of the evaluated models correspond to those found excluding the C-terminal domain.

c Regarding to AlphaFold structure. The RMSD values were calculated considering 272 pairs of residues.

d Experimental structures of GPR40 in inactive states.

e Experimental structures of GPR40 in active states.

Free energy landscape (FEL) calculations were performed to evaluate the effect of the water model on the receptor configurational changes during MD trajectories. Taking the RMSD and radius of gyration as variables, free energy associated with each configurational substate was calculated, both for the complete structures and those without the C-terminal domain. The results revealed that the structures with the most significant distribution of energy substates were those simulated with the rigid models (0.086 and 0.091 nm^2^, respectively). Furthermore, the structures with the highest energies were the G40-spce systems (14.8 kJ/mol), with two structures with the lowest energy in frames 280.2 and 333.6 ns (Figure 2c). On the other hand, for the G40-fba and G40-t4 models, the maximum energies were 12.8 kJ/mol. The G40-fba exhibited three minimum energy configurations at 34.2, 191.8, and 392.6 ns, while for the G40-t4, two clusters with five energy minima at 170.8, 347.8, 358, 389.6, 486.4, and ns. In the case of the TIP4P/ϵ_flex_ model, the lowest distribution of energy states was observed (0.078 nm^2^) with maximum energies of 13.0 kJ/mol. However, this system showed a greater distribution in minimum energy states than the other models, with two minimum structures at 412.2 and 470.4 ns. Using a similar analysis, Sharma et al. obtained an energy range of 0–8.88 kJ/mol for the apo form of this receptor interacting with the SPC water model.^22^

With the purpose of quantifying the energy contribution and the distribution of substates caused by the movements of the C-terminal domain, the same analyses were repeated without this domain, and the FEL maps were compared (Figure S4b,c). The results found were notable. As expected, the number of configurations is reduced in all water models, and the minimum energy clusters are more focused, reducing the number of metastable substates. The G40-t4 and G40-fba systems presented a decrease in the distribution of substates close to 86%, while G40-spce and G40-t4f a 70%. Regarding energies, the system showing a more significant reduction in the maximum energy structures was G40-fba, with a decrease of 1.8 kJ/mol. In contrast, the G40-t4f structures only decreased by 0.3 kJ/mol. Based on these results, the SPC/E and TIP4P/ϵ_flex_ models exhibited the greatest interaction with the TM domains of the GPR40 receptor, which would imply a greater capacity to modify the active/inactive states of the receptor. However, this also suggests that the C-terminal domain could play an important role in receptor interactions in the intracellular region of the cell.

### Four Water Models Lead the GPR40 Receptor to an Active-Like State Configuration

The biological function of a protein is related to its native conformation or close to it.^82^ In the absence of ligands (apo form), the lowest energy structures are expected to represent the native conformation of the receptor. Due to this, to explore the structural changes of each model, the minimum energy structures of each trajectory were extracted. In the case of multiconfigurational minima, the structure chosen was the one with the longest simulation time (Figure 2d). As previously discussed, a displacement of the TM domains outward in all structures is observed, both in the extracellular and intracellular regions concerning the initial configuration. This open conformation is accompanied by a loss of α-helix structures, evidenced by the breakage of the helical tubes used to represent these domains (Figures 2d and S5). The most significant opening and loss of secondary structure is observed in the G40-t4f model.

Noteworthy, all four structures feature the opening of the TM5 and TM6 domains in the intracellular region. Prior research has demonstrated that the outward movement of the TM6 domain is a significant feature of the transition to active states in GPCRs.^15,33^ As mentioned by Katritch et al., the outward kink initiates around the highly conserved residue P239 (Proline^6.50^ in the TM6 domain).^15^ However, the most significant bend angle in our structures occurs between residues T198-R211 in TM5 and H216-A229 in TM6.

**Figure 3:**
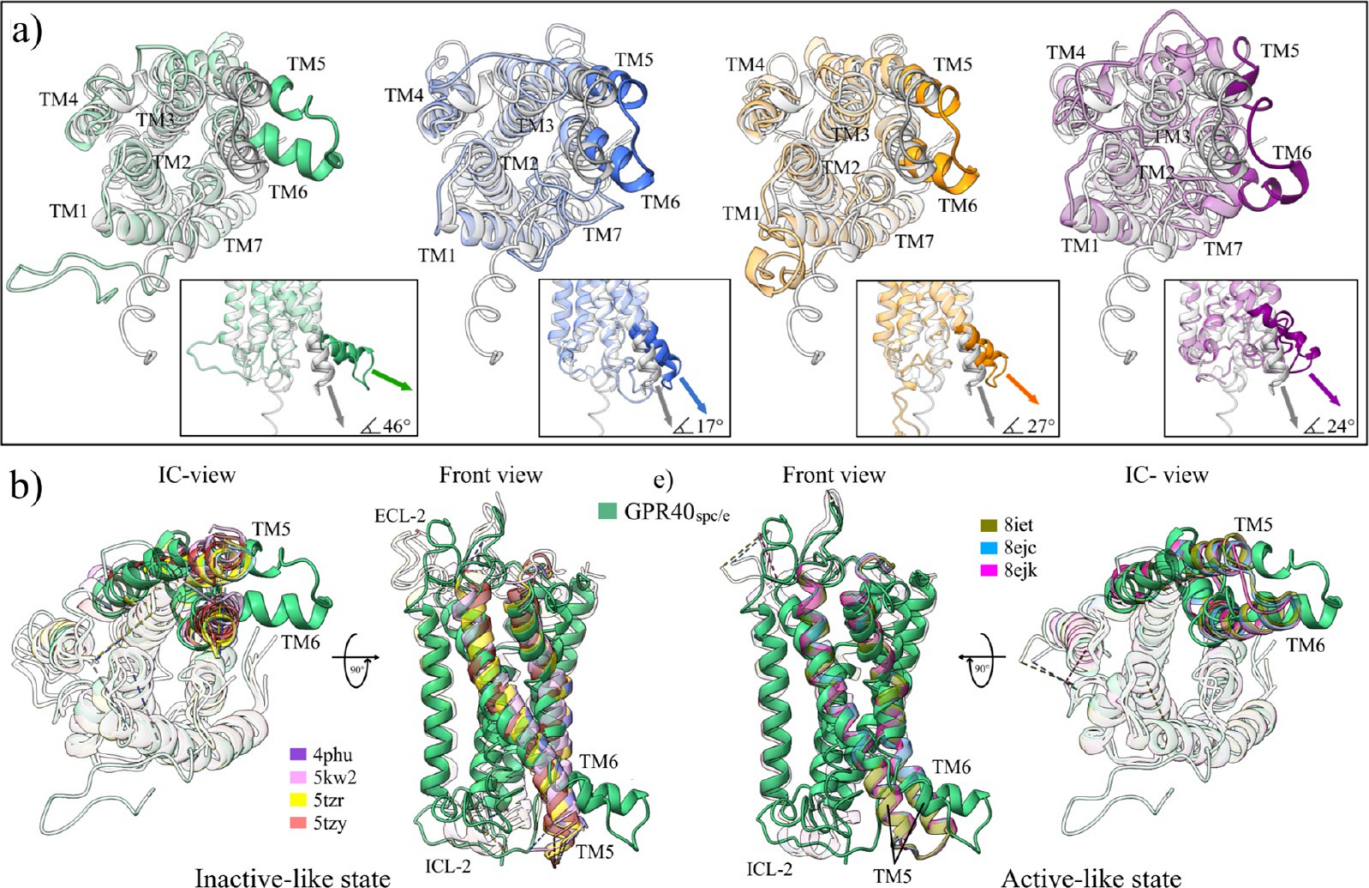
Structural alignment with the active forms obtained at the end of MD simulations. (a) Bending angle of the TM5 and TM6 domains in the intracellular region, taking the AFG40 model as reference. The angle was measured based on the centroid of the helices of residues T198–211 in TM5 and H216-A229 in TM6. (b) Comparison of the GPR40 system - SPC/E water model with experimental GPCR structures in inactive and active states. The figures highlight the ICL-2, TM5, and TM6 domains, which are considered indicators of the activation state in these receptors.

As can be seen in Figure 3a, this “hinge” type movement and the bend angle were different in the four models studied. For the G40-spce structure, both domains (TM5 and TM6) were raised at an angle close to 46° concerning the initial conformation. This angle increases if compared to the structure in an inactive-like state 4phu, reaching a value of 57°. In the G40t4 model, the elevation angle was the lowest observed, 17°(28°), being the model that preserved the helical structure of both domains. For flexible models, the bend angle was 27° (38°) for G40-fba and 24° (35°) for G40-t4f. In this last model, the loss of the helical structures is clearly observed. Finally, the four structures lose the helical structure of the ICL-2 domain, which several authors relate to active conformations. This domain is stabilized by four hydrogen bonds, F110-Y114, L112-Q115, G113-A116, and Q115-R118 in the initial structure. Only the G40-t4f structure conserves part of this domain, which presents two new interactions, Y114-F117 and Q115-R119, added to the conserved Q115-R118.

Despite the exciting nature of these findings, it is necessary to comment that the increasing presence of water molecules exacerbates this “hinge” type movement on intracellular TM5 and TM6 domains and the loss of the helicoidal form of the ICL-2 domain. The structural properties and parameters of the models used allow protein–water interactions to be strong enough to cause a hydrophobic effect on the receptor (Table 1). Furthermore, it has been shown that helix-like structures are not favored in environments with high electrostatic interactions.^9^ Although no evidence supports whether this is a deficiency of the water models used, the loss of helical structure in the systems would go in that direction.

As observed so far, the direct effect of the four water models is to drive the GPR40 structure to an apparently active state. The MD trajectories show a loss of secondary structure, opening of the TM domains, and high activity of the C-terminal domain. To better understand these changes, a comparison has been made with the seven reported experimental structures of the GPR40 receptor. Other GPCR structures were also used for this purpose: 3vw7^83^ in inactive state and, 5nx2,^84^ 2rh1,^77^ 5g53,^85^ 3sn6,^86^ and 4zwj^78^ in active state. Results are shown in Figures 3b, S6, and Table 3.

By superimposing the structures of the GPR40 receptor in the inactive and active states against those obtained in our simulations, the results support the idea that the water models used in this work tend to form configurations similar to the active ones. Figure 3b shows the structural overlap of the G40-spce model in which its high bend angle of the TM5 and TM6 domains described above stands out. When calculating the RMSD in the sequence alignment, a more significant overlap is observed with the structures in the active state than inactive ones (3.07 versus 3.89 Å). This trend is repeated with the other configurations obtained in this work (Figure S6 and Table S1). Notably, the experimental structures with the highest overlap, both in the inactive and active states, were those that formed complexes with the drug TAK-875 (4phu and 8ejk).^31,32^ Furthermore, the G40-fba model had the highest overlap regarding the GPR40 experimental structures. The average values obtained for this model were 3.06 and 2.76 Å for the inactive and active states, respectively.

Another important structural feature observed in both the lowest and final energy conformations is the loss of the secondary structure of the receptor with the four water models. Columns 3–6 of Table 3 show the analysis and comparison of these structures with various experimental models. For this purpose, the AF40 structure was taken as a reference, composed mainly of a helical structure (71%) and two β-strands formed by residues I160–V140 and S167–C170. About the latter, the only model that loses these structures is the G40-fba. In the case of the G40-spce and G40-t4 systems, the four residues reported experimentally formed these β-strands. For model G40-t4f, an additional β-strand was formed between residues H153–N155.

On the other hand, the loss of helical structure is dramatic in the four models studied. The G40-t4 and G40-fba models were the ones that conserved a higher percentage of these structures (58.0 and 58.6%, respectively), with the second being the one that formed a higher rate of 3–10 α-helix. The most significant loss of helical structures was obtained by the G40-t4f model, retaining only 44% of them. These results would reinforce our idea that the interactions of the SPC/E and TIP4P/ϵ_flex_ models with the receptor are stronger than those of the other two models, which triggers a more significant loss of the secondary structures.

### Correlation between Water Properties and Configurational Changes of GPR40-Membrane Systems

The conformational changes observed so far are highly dependent on the environment surrounding the GPR40 receptor and, to a greater extent, on the solvent used. According to the model proposed by Frauenfelder et al., protein movements at long scales are dominated by solvent bulk fluctuations, while at short scales, they are due to the hydration layer.^87^ Although studying the latter is vital to understanding the biological function of this class of receptors, this work focuses only on analyzing those properties due to interactions with bulk water.

#### Root Mean Square Fluctuations and Diffusion Coefficient

**Figure 4:**
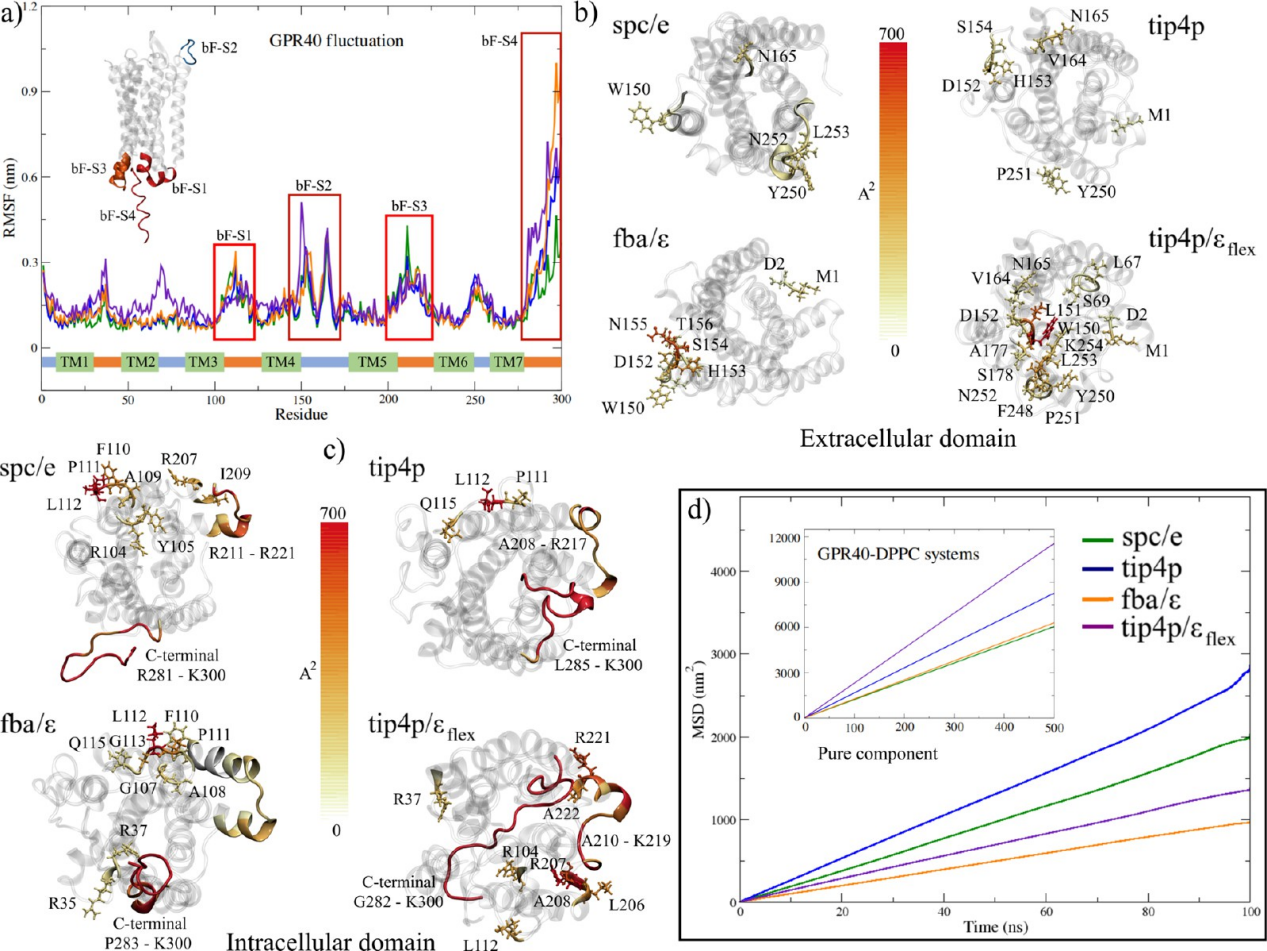
Analysis of the fluctuations per residue of the GPR40 structures. (a) Plot of root-mean-square fluctuation (RMSF) by residue. The 3D structure in the box indicates the location of the high-fluctuation zones. (b) Residues with greater fluctuation in the extracellular region of the receptor, and (c) in the intracellular or cytoplasmic region. (d) Average MSD of the water models in the GPR40-DPPC systems.

The fluctuations of the GPR40 residues were calculated using the RMSF descriptor, which allows for analyzing local changes, flexible regions, or interacting sites. The results revealed that the four solvent models exhibit three regions with oscillations close to 0.6 nm and one more with greater than 0.6 nm (Figure 4a).

The sites of medium fluctuation (bF-S1 and bf-S3) correspond to the ICL-2 domain and the region that forms the “hinge” in the intracellular part of the TM5 and TM6 domains, both associated with the active/inactive states in GPCRs (Figure 4c). On the other hand, the bf-S2 site belongs to the ECL-2 domain, which is part of one of the extracellular allosteric sites mentioned by Srivastava et al.^32^ These authors point out that ECL-2 acts as the roof of the pocket where TAK-875 ligand binding occurs, mentioning the high stability of this domain (34–88 Å^2^ in B-factor). Interestingly, this site presented high fluctuations in all models, with residues G149-T156 and P163-G166 being those with the highest molecular vibration observed (Figure 4b). At this site, the systems solvated with the flexible models achieved higher fluctuation, reaching values close to 0.6 nm (1552.05 Å^2^) in the G40-t4f model. These results suggest that the high fluctuation at this site is due to the absence of the TAK-875 ligand so that the allosteric pocket could be in a partially activated state. Finally, the maximum fluctuation per residue was located in the C-terminal domain (bF-S4), mainly with the FBA/ϵ model. This result was expected, given the results obtained previously. Figure 4c shows those residues that reached the highest values of B-factor in the intracellular region.

The high fluctuation of extracellular and cytoplasmic domains has been mainly related to the dynamic properties of water in several studies.^5,88^ A property that has been studied to understand protein–solvent interactions, flexibility, and activity in proteins is the diffusion coefficient.^89^ However, given the complexity of the interactions, the dependence on distance, and the fact that it becomes anisotropic at the interface with the protein, our RMSF results were compared to the self-diffusion of water as a pure component (Figure 4d).

The values of the self-diffusion coefficient (D) exhibit an overestimation of the diffusive behavior of water in the SPC/E, TIP4P, and TIP4P/ϵ_flex_ models concerning that experimentally estimated at 309.65 K, especially in the four-site models (Table 4). For TIP4P, the overestimation was 44.85%, while for the flexible model, it was 27.24%. In the case of the FBA/ϵ model, an underestimation in the value of *D* close to 46% is observed concerning the experimental value. It is interesting to note the low diffusion of this last model because it is one of the systems that showed high fluctuation in the receptor structure (0.16 ± 0.13 nm, Table 2). However, these results agree with what was observed regarding the loss of secondary structures, which suggests that low diffusion would better preserve helical structures. This overestimation is also observed by Ferrario and Pleiss, who evaluated this property in the soluble enzyme CALB (Antarctic Candida Lipase B) using the SPC/E, TIP3P, and TIP4P models at 300 K.^90^ In order to compare the diffusion of the models as a pure component, this property was calculated in the GPR40-membrane systems (inset of Figure 4 and Table 4). The calculation was carried out in three dimensions without considering the diffusion processes of the hydration layers. It can be seen that the G40-t4f system was the one with the most significant diffusion, reaching an overestimation of 50.5%. This high diffusivity was expected, given that the most notable structural changes of the receptor were obtained in this system.

**Table 4:** Calculated Self-Diffusion Coefficients, Densities, and Dielectric Constants for the Four Water Models Used in This Work (*T* = 309.65 K)^a^

property	water model				refs (90 and 91)^c^			exp^92,93^
	SPC/E	TIP4P	FBA/ϵ	TIP4P/ϵ_flex_	SPC/E	TIP3P	TIP4P	
self-diffusion coefficient (×10^–05^ cm^2^/s)	2.01 ± 0.01	2.75 ± 0.02	2.09 ± 0.01	4.53 ± 0.03	2.91	6.29	4.00	3.01
	*3.29 ± 0.12*	*4.36 ± 0.19*	*1.64 ± 0.02*	*3.83 ± 0.14*				
density (kg/m^3^)	1000.0 ± 1.4	995.6 ± 1.5	985.5 ± 1.4	967.5 ± 1.4	988.9	974.0	982.9	993.51
	*992.9*	*986.8*	*995.0*	*972.5*				
dielectric const.^b^	72.2 (41.9)	77.8 (31.5)	71.35 (40.6)	80.1 (44.1)	72.3	98.7	51.3	74.37
	*68.9*	*49.6*	*71.9*	*77.3*				

a The values in italics correspond to simulations as a pure component of the four water models considering 500 molecules at 309.65 K and 1 bar of pressure (second row for each model).

b The values correspond to the dielectric constant obtained considering all the system components and, in parentheses, only the water molecules.

c Values reported at 300 K.

On the other hand, diffusion processes are also important in protein–membrane systems. Lateral diffusion of integral membrane proteins and lipids plays a vital role in their biological functions.^94,95^ Necessary for correct homeostasis, diffusion promotes the interaction of proteins with other components, increases the distribution of accessible states, or amplifies the response to a perturbation.^96^ The lateral diffusion of both the lipids and the protein was calculated to characterize their behavior. In the case of lipids, the displacement of the phosphorus atom (P8) was taken as a reference, while for the protein, the center of mass was followed. The diffusion coefficients were obtained by calculating the MSD perpendicular to the *z*-axis.

**Figure 5:**
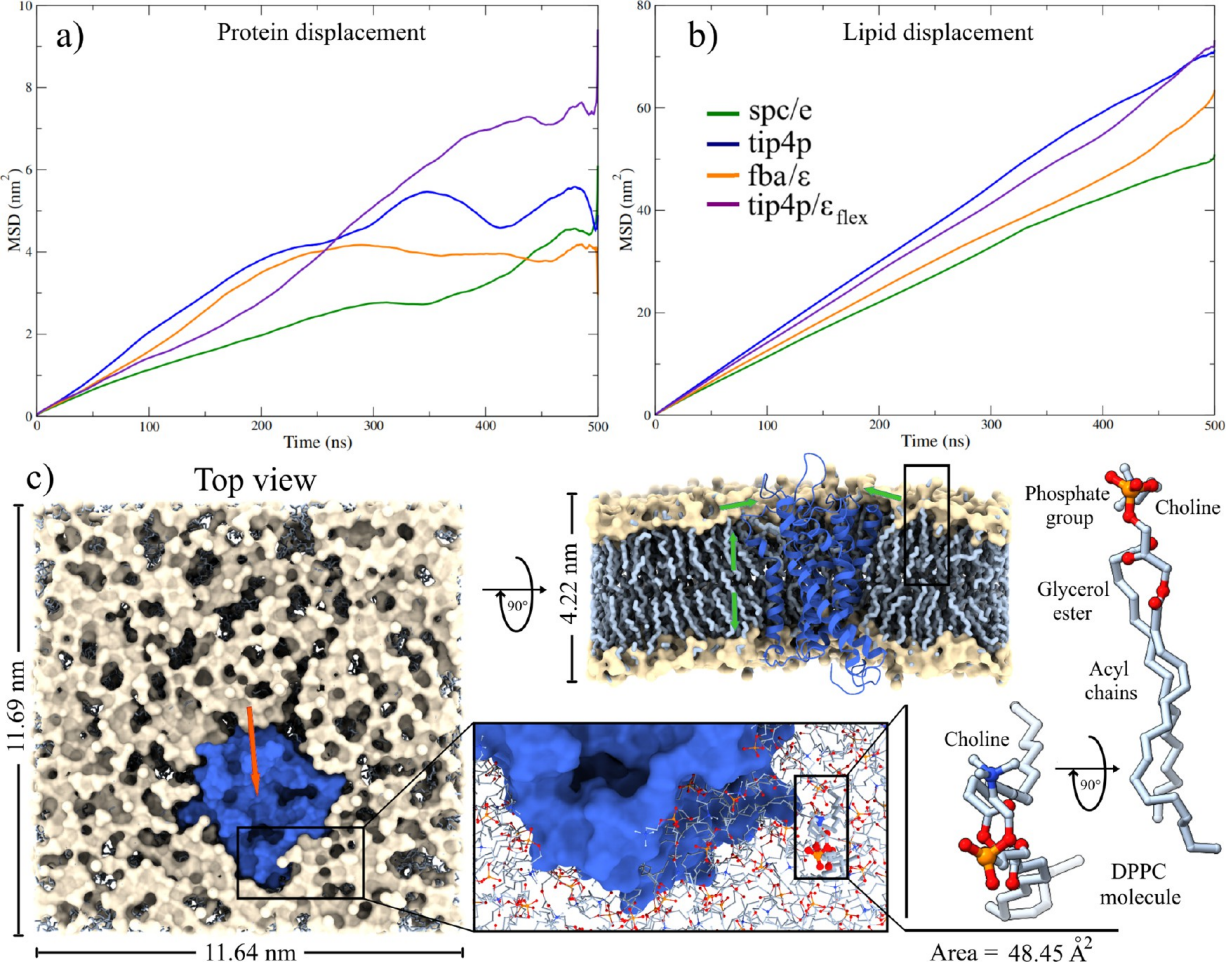
Lateral Mean Square Displacement plots on the *z*-axis. (a) GPR40 receptor. (b) Lipid molecules. (c) Effect of lateral diffusion of the receptor and lipids on the structural configuration of the GPR40-membrane-TIP4P complex. The compaction of lipids and their interaction with the receptor causes a decrease in the membrane area and the displacement of GPR40 (orange arrow, left panel). This decrease in area per lipid is observed in the alignment between the acyl chains (bottom and right central panel), increasing the thickness of the membrane. Green arrows indicate the movement of lipids near the receptor structure (top center panel).

Figure 5a shows the diffusive behavior of the GPR40 during the simulation trajectories. It can be seen that in the two flexible models and the TIP4P, the displacement of the receptor is greater than that in the case of the SPC/E model. However, in the last 250 ns of simulation, there is a subdiffusive behavior in the FBA/ϵ and TIP4P models with notable perturbations in the latter. This anomalous subdiffusion suggests more significant interaction with membrane lipid molecules, hindering lateral diffusion of the receptor in these models. Statistically, this can be observed in the increase in the standard deviation in the calculation of this property (Table 5). Such unstable subdiffusive behaviors have been observed in other GPCR structures.^97,98^ Of the four models, SPC/E is the one that showed the most stable and constant movement of the receptor through lipid molecules. It is important to note that these parameters of the DPPC membrane were taken from the work carried out by Tieleman and Berendsen, who used the SPC and SPC/E water models to obtain them.^60^ In the case of the TIP4P/ϵ_flex_ model, superdiffusive behavior was observed starting at 200 ns. Although discordant with the other models, it is the system that came closest to the reported value of the diffusion coefficient obtained by Ramadurai et al. for membrane proteins with radii of gyration close to those observed in our analyses (∼1.3 nm, *D*_prot_ in Table 5).^94^

**Table 5:** Physical Properties of the DPPC Lipid Bilayer at 309.65 K^a^

water model	diffusion (×10^–07^cm^2^/s)		area per lipid (Å^2^)		bilayer thickness *D*_HH_ (nm)
	protein	DPPC	up	down	
exp.	0.50^94^	0.38^99^	51.68^100^		3.6–4.4^101^
SPC/E	0.18 ± 0.09	2.58 ± 0.24	47.26 ± 0.31	46.12 ± 0.91	4.35 ± 0.10
TIP4P	0.25 ± 0.36	3.65 ± 0.44	47.82 ± 0.11	47.10 ± 0.05	4.21 ± 0.08
FBA/ϵ	0.19 ± 0.48	2.82 ± 0.36	47.99 ± 0.33	47.20 ± 0.36	4.25 ± 0.07
TIP4P/ϵ_flex_	0.46 ± 0.22	3.43 ± 0.09	48.81 ± 0.66	47.33 ± 0.71	4.38 ± 0.02

a The experimental values reported are at 309 K. For the area per lipid, Up is the top layer and Down is the bottom layer.

**Table 6:** Key Resources Table

software or server	source	identifier
Gromacs v.2021	(56)	https://manual.gromacs.org/2021/download.html
VMD	(69)	https://www.ks.uiuc.edu/Research/vmd/
UCSF ChimeraX	(119)	https://www.cgl.ucsf.edu/chimerax/download.html
UCSF Chimera	(70)	https://www.cgl.ucsf.edu/chimera/download.html
RCSB PDB	(36)	https://www.rcsb.org
UniProt	(35)	https://www.uniprot.org
AlphaFold DB	(48,120)	https://alphafold.ebi.ac.uk
DeppTMHMM	(55)	https://dtu.biolib.com/DeepTMHMM

#### Effect of Lateral Diffusion on Lipid Molecules

MSD analysis of the lipid molecules exhibits a normal diffusion process (Figure 5b). All systems exhibit slight disturbances in the last 150 ns of simulation, which is more evident in the G40t4 system. Furthermore, the four-site models are the ones that present the greatest diffusivity, with the flexible model being the one that showed the most significant displacement. The diffusion coefficients obtained (*D*_lip_) overestimated 1 order of magnitude concerning the experimental value in DPPC membranes,^99^ although they were similar to other in-silico studies.^102^

Several structural properties of membranes are closely related to the diffusion process of their molecules, among which are the area per lipid and the thickness. The lipid structure can determine vital processes, such as the activity or biological function of the receptor, its orientation, and interactions.^103−105^ In this work, the area per lipid was calculated using the GridMAT-MD program.^106^ The thickness of the membrane was determined by measuring the distance between the points of the highest density of the polar heads of the lipids.

The effect caused by the diffusion of the different components of the studied systems can be visualized when analyzing the final structures. Figure 5c illustrates this fact, taking the G40-t4 system as an example. At the beginning of the simulations, the structural parameters of the lipid bilayer are the experimental values observed for the DPPC membrane type (area per lipid = 60.5 Å^2^ and thickness = 3.98 nm).^79−81^ Nevertheless, these values correspond to the membrane at 323 K. In this work, the simulations were carried out at body temperature (309.65 K), so a change in both properties would be expected. For the TIP4P system, at the end of the trajectories, the area per lipid decreased by 21.6%, and the thickness increased by 5.8%. These structural changes in the membrane direct the lateral displacement of the receptor, displacing it from the central area of the simulation cell (orange arrow in Figure 5c, left panel). However, as the area per lipid decreases, the movement of the protein slows down, causing a decrease in its diffusion through the membrane (bottom center panel and right panel of Figure 5c). Lipid compaction increases bilayer thickness and neighboring lipids of the receptor to cover a greater area of the hydrophobic surface of the receptor (green arrows in the upper center panel of Figure 5c). The latter explains the change in the solvent-accessible surface area (SASA), which decreases from an initial value of 162.37 to 157.39 nm^2^ at the final MD trajectories in the case of the TIP4P model (Table 2). A similar behavior is observed in the remaining systems (Figure S7).

For the area per lipid, the values of both layers are reported in Table 5. In the extracellular region, the values are slightly higher because that layer has one less molecule than in the intracellular layer (254 and 255, respectively). In the four systems, similar average values were observed, lower than those reported by Walter et al.^100^ Using machine learning techniques, these authors could determine this property at different temperatures, including 310 K. On the other hand, regarding the thickness of the membrane, the values are within the range reported by Leonenko et al., who obtained them experimentally by scanning force microscopy.^101^ Although diffusion is not entirely represented accurately in these systems, the structural properties fall within the experimentally obtained parameters.

#### Solvent Accessible Surface Area (SASA) and Density

During the analysis of the diffusion process, it was observed that the systems exhibit similar structural behavior concerning the membrane. However, in the TIP4P/ϵ_flex_ system unlike the others, an increase in membrane thickness does not result in a decrease in SASA. Contrary to what was expected, the calculated value of SASA was 165.58 nm^2^, representing an increase of 1.9% compared to the initial value. Furthermore, our previous analyses suggest that this water model has a greater interaction with the receptor atoms, leading to the instability of its structure. It is known that the presence of water at the interface of macromolecules can alter the network of intramolecular interactions, leading to their denaturation.^107^ With this idea in mind, the calculation of the partial density profiles of the receptor and the different groups of the membrane was carried out in order to detect water molecules in their vicinity.

**Figure 6:**
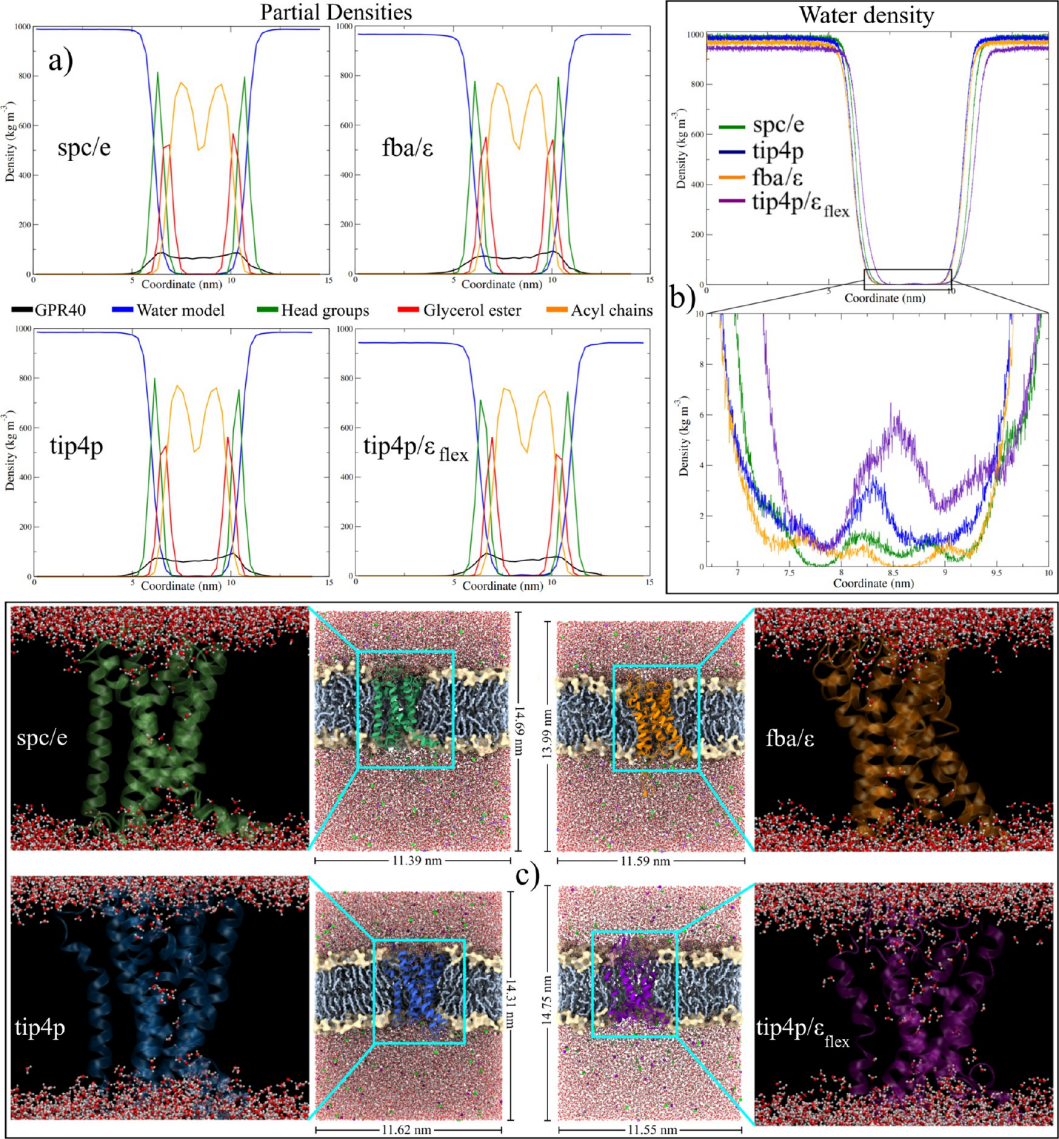
Density analysis of the systems studied. (a) Partial density profiles of the different groups in the GPR40-membrane-water systems. (b) The water density profiles indicate the presence of water molecules in the core of the receptor. (c) Distribution of water molecules in the transmembrane region of the systems. The images correspond to the last frame of the MD trajectories (500 ns).

In density profile calculations, lipids were divided into three groups: the two acyl chains (hydrophobic region), the glycerol ester group, and the polar head (polar region). The phosphate and choline groups form the latter. The density of the protein and the solvent were also evaluated. Figure 6a depicts the graphs of each system. Overall, the membrane exhibits robust structural stability because the profiles of each group considered are preserved, and both lipid layers retain their symmetry. However, subtle differences are found in the evaluation of each group (Figure S8). The average density and thickness values for lipid tails were 762 kg/m^3^ and 2.03 nm, respectively. The average values for the glycerol ester group were 543.8 kg/m^3^ and 3.25 nm, while for the polar heads, the average values were 775.3 kg/m^3^ and 4.34 nm. The lowest densities occurred in the TIP4P/ϵ_flex_ system, meaning a greater distribution of lipids along the *z*-axis and, consequently, a greater membrane thickness. These values are consistent with previous in-silico studies, which used the DPPC membrane and SPC, SPC/E, and TIP4P water models at temperatures close to 310 K.^108−112^

Regarding the receptor structure, the maximum density values represent the solvent-membrane interface in the intracellular and extracellular regions (left and right zones of the graphs, respectively. Figure 6a). When analyzing the different curves (Figure S8, lower right panel), it is observed that the density of the G40-spce and G40-t4f models is high in the intracellular region and decreases to a greater extent in the extracellular region of the G40-t4f receptor. The opposite situation occurs in the G40-fba and G40-t4 models. The explanation for these density values in the intracellular region is due to the interactions of the C-terminal domain. While in the G40-spce and G40-t4f structures, this domain interacts with both the lipids and the TM domains, causing it to fold toward them, in the other two models, this interaction is transient, remaining unfolded for much of the simulation. On the other hand, the extracellular region is where the most significant loss of secondary structure occurs in the G40-t4 model, decreasing its density. On the contrary, these structures are well preserved in the rest of the models, so high-density values are observed.

The unclearest result was obtained by calculating the density of water in the systems. Although a decrease in density was expected due to the presence of the different components, the values showed a significant reduction, especially in the flexible models. Table 4 displays the density values obtained using the Gromacs *gmx energy* tool. However, the calculated value represents the total density of the system, which differs from the values calculated with *gmx density*. With this tool and dividing the simulation box into 1000 slices, the values obtained were 988.3 ± 5.5 (SPC/E), 983.8 ± 5.6 (TIP4P), 966 ± 6.5 (FBA/ϵ), and 943.5 ± 5.2 (TIP4P/ϵ_flex_). The experimental density at physiological temperature is 993.52 kg/m^3^, so this discrepancy is notable. When comparing the different profiles, it was observed that part of the water molecules entered the core of the receptor (Figures 6b and S9), which has been observed in the GPR40 structure and other GPCRs.^34,83,85^ Various authors even mention that water molecules help stabilize the GPCR structures, activate them, and participate in their active sites.^113,114^ However, the density in the transmembrane region is not significant enough to explain the decrease in bulk density. Even more, the highest density in this region was for the two four-site models, which would partially explain the G40-t4f system but not that of the G40-fba model, producing the lowest density observed.

However, an important fact could be inferred from the analysis of the penetration of water molecules into the receptor core. As seen in the lower panel of Figure 6b, the TIP4P models included a larger number in the transmembrane region. When taking a snapshot of the last frame of the simulations, it was observed that the molecules of the G40-t4f model were very dispersed over the TM domain. This could explain why the structure of this system presents the most remarkable configurational differences compared to the other models. In this system, the interaction force of water is large enough to cause the loss of structure of the receptor. Water molecules are even observed penetrating the membrane. By contrast, the three-site models exhibit a more ordered intrusion of the molecules, especially in the extracellular region of GPR40.

##### H-bonds and Dielectric Constant

**Figure 7:**
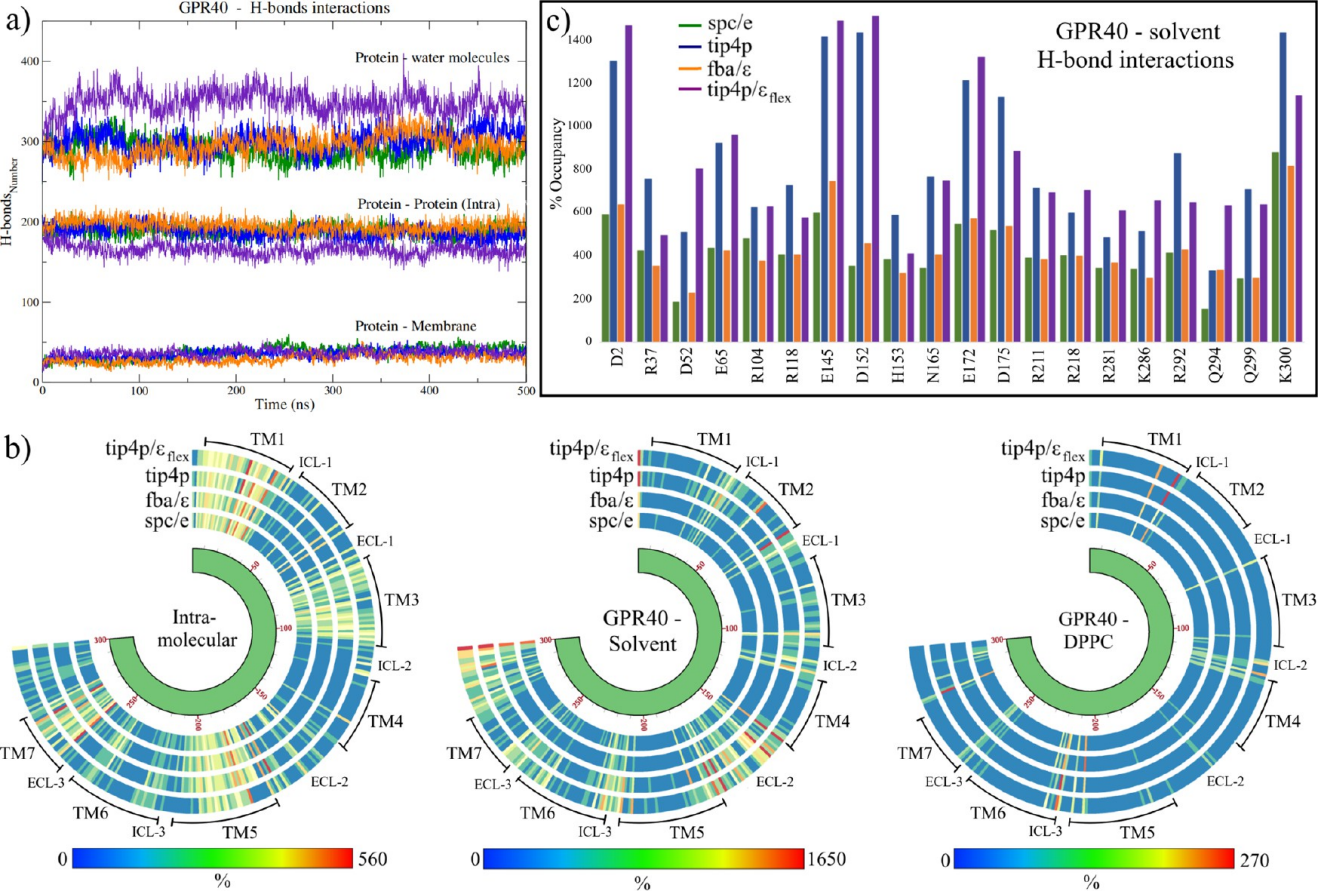
Analysis of hydrogen bond interactions. (a) Number of H-bonds formed throughout the simulations between the GPR40 and the different components of the system. (b) Heat map of the occupancy percentage per residue of the interactions analyzed. Each circle in the diagram represents the entire receptor sequence, while each tick represents an individual residue. (c) Top 20 residues with the highest percentage of H-bond occupancy in GPR40-water molecules interactions.

The effect of water models on the structural properties of the GPR40 receptor is evident. Optimized with different interaction parameters (Table 1), these models impact the flexibility, motion, and stability of the receptor. Experimentally, it is known that upon solvation, proteins can contain a layer of water molecules with a thickness close to 5 Å, known as the hydration layer (HL).^11^ Being dynamic, the HL continually exchanges molecules with the protein and the bulk, with hydrogen bonds (HB) being the most relevant interactions on the protein surface.^115^ These interactions play an important role in the stability of secondary and tertiary structures and affect the folding or denaturation processes in proteins.^5^ As they are essential interactions in studying these systems, we have evaluated the formation capacity of these interactions in the four models using the Gromacs *gmx hbond* tool. The intramolecular HB of the GPR40 (HB_intra_) and those formed by its interactions with the solvent (HB_sol_) and lipids (HB_lip_) were evaluated (Figure 7 and Table 2).

Based on the results, it is evident that the G40-t4f structure experiences high instability. The instability can be attributed to the water molecules in this model, which have the remarkable ability to break the intramolecular hydrogen bonds of the receptor and “hijack” them to the solvent. This is evidenced by the significant decrease in HB_intra_ (−13.2%) and the increase in interactions with the HB_sol_ (+17.2%) compared to the average values of the other three models. Consequently, there is an enlargement in the solvation layer (SASA values), loss of secondary structure, and an overall increase in molecular fluctuation and volume. Regarding the HB_lip_, the G40-fba structure is the one that had the weakest interaction with the membrane molecules. However, the latter results do not allow identifying those domains or residues involved in forming HBs. In this sense, to determine the specific contribution of each residue, we calculate its occupancy percentage. This is the fraction of the simulation time in which any of its atoms participate in the interaction. The obtained percentage values correspond to the total number of interactions observed for each trajectory. Since each residue can have multiple atoms participating in these HBs, the occupancy percentages are usually high.

The left panel of Figure 7b depicts the heat map of the per-residue occupancy values in the intramolecular interactions. Remarkably, all four models show that HB formation is concentrated in the TM1, TM3, TM5, and TM7 domains, with TM1 being the region with the highest activity in these interactions. The residues with the highest occupancy in the four systems were N23, I27, H33, R183, R258, and N272, located in the extracellular region of the TM1, TM5, and TM7 domains. Interestingly, residues R183 and R258, together with N244, are part of the allosteric site of GPR40, which interacts with both endogenous and synthetic ligands.^32,116,117^Table S2 shows the top 20 residues with the highest values in HB_intra_ occupancy. It should be noted that the ECL-2 domain exhibits HB formation, which helps to stabilize the receptor. Within this domain, residue E172 displays high occupancy percentages in the G40-spce, G40-t4, and G40-fba models. Experimental findings suggest that this residue keeps the receptor in its inactive state by interacting with residues R183 and R258 while also serving as a roof for the allosteric site mentioned earlier.^32,118^ Finally, it is observed that the loss of the helical structure of the G40t4f model occurs in the TM5, TM6, and TM7 domains. Additionally, this water model is the only system that maintains the helical shape of the ICL-2 domain, stabilized by the formation of HB_intra_.

Regarding interactions with the solvent, a high formation of HB_sol_ is observed with the TIP4P and TIP4P/ϵ_flex_ models, especially in the ECL-2 and C-terminal domains (Figure 7b, middle panel). The occupancy in these domains reached values greater than 1000% (E145, D152, E172, D175, and K300, Table S3). Other residues with high occupancy were M1, E65, and R292. It is important to note that at all nontransmembrane sites, the four-site models interact to a greater extent than the three-site models. In relation to the high formation of HB_sol_ in the ECL-2 domain, Srivastava et al. mention that this domain is stabilized mainly by different polar interactions, among which are those formed with water.^32^ Furthermore, they report that the only area with high flexibility is located between residues D152 - N155, which is also observed in our results. However, Kumari et al. point out that this domain is the orthosteric binding site of the endogenous ligands γ-linolenic acid (γLA) and docosahexaenoic acid (DHA).^31^ Noteworthy, if the percentages of the residues with the highest occupancy are compared, notable differences are seen between the four- and three-site models. According to the values obtained, the HB_sol_ formation and the time they are preserved are significantly higher in the TIP4P models (Figure 7c).

To a lesser extent, the HB_lip_ formed by the interaction with the lipids is similar in the four models. Domains TM1, TM5, and TM6 are the ones that showed the highest occupancy (Figure 7b, right panel). Remarkably, the results indicate a high HB_lip_ formation on residues R207 and R211 in TM5 and H216, R217, K219, and R221 in TM6 (Table S4). Located at the intracellular “hinge,” these HB_lip_ interactions could contribute to the opening of these two domains to lead GPR40 to its active state. Worth mentioning is the formation of HB in the ICL-2 domain with both lipids and water molecules. This region is crucial in the receptor interactions with downstream effectors.^33^ Being one of the sites that displayed high fluctuation in the simulations, the results suggest that this region maintains a high interaction capacity even in the absence of ligands.

On the other hand, the formation of HB between the membrane and the solvent was also evaluated. The analysis revealed that the TIP4P models interacted more strongly with the polar region of the lipids as compared to the FBA/ϵ and SPC/E models (Figure S10 and Table 2). On average, the number of HBs formed in these models exceeded that of the FBA/ϵ and SPC/E models by 4.5 and 11%, respectively. Experimentally, it has been demonstrated that the structural properties and lateral diffusion of membrane components are influenced by the amount of water penetrating the interface.^12,13^ Our density analyses showed that the four-site models had a higher penetration level than the three-site models, which could explain the almost double difference in the diffusion coefficients observed in the TIP4P and TIP4P/ϵ_flex_ systems.

Finally, the formation of HBs in the solvent was analyzed in an attempt to correlate it with the electrostatic properties of the systems. In order to compare these analyses with the general properties of the four models, these properties were also calculated in water as a pure component. The results were notable and are shown in Table 4 and Figure S11. As a pure component, the dielectric constant values were as expected. The flexible models were the ones that best reproduced this property with errors of 3.3 (FBA/ϵ) and 3.9% (TIP4P/ϵ_flex_), while the rigid models had errors of 7.4 (SPC/E) and 33.3% (TIP4P). However, the formation of H-bonds did not correlate with dielectric constant results. As shown in Figure S11a, it can be seen that the four-site models form four times more HBs than the three-site models. This is explained by the higher atomic charge of the four-site models (Table 1) and, to a lesser extent, by the flexibility of the models.

Interestingly, the formation of HBs in the systems studied differs entirely from what was observed as pure components (Figure S11b). In these systems, the rigid models exhibited a greater number of HBs, and the G40-t4f system formed the least. The G40-fba model had intermediate HB values. These results suggest that as there are more components in the simulated system, the flexibility of the model becomes more relevant in the formation of HBs in the solvent. In the presence of nonregular surfaces due to the receptor and membrane structures, a flexible model can interact better with them. This can be seen in the values in Table 2, where the flexible models form more HBs than the rigid models. Furthermore, the flexibility could reduce the probability of HB formation in the solvent due to the geometric parameters imposed when quantifying these HBs. This flexible feature could be an advantage when studying these biomolecular complexes.

As in the pure component case, the dielectric constant does not correlate with what is observed in the formation of HBs. If the complete system is taken for its calculation, a drastic change is observed in the G40-t4 model, similar to that exhibited in the diffusion calculation. At the end of the simulation, it is observed that the systems, in general, reach values similar to that of water as a pure component. On the other hand, if only the solvent is considered, the dielectric constant decreases, although it maintains a behavior comparable to that of the pure component. However, our calculation has two critical limitations: considering all the system components and the simulation cells’ volume. The dielectric constant is a property that depends on the direction in which it is measured, and in inhomogeneous systems, its calculation does not cause problems. In the case of our systems, heterogeneity limits the adequate description of this property, resulting in a slow convergence in its analysis. In contrast, if only the solvent is taken in its calculation, the poor description of the volume occupied by the molecules generates an underestimation of their values.

### Conclusions

In this work, we tested four water models to describe the GPR40 interactions on membrane-solvent systems, namely, SPC/E, TIP4P, FBA/ϵ, and TIP4P/ϵ_flex_. Our analysis revealed that all four models had certain deficiencies in the representation of the GPR40 structure. The most notable was the loss of the helical structure of the TM domains due to the strong interaction of water molecules with the receptor residues, which is most evident in the SPC/E and TIP4P/ϵ_flex_ models. Consequently, it induces the receptor to acquire active configurations on the intracellular domains of TM5 helices, forming a “hinge” type structure between residues T198–211 in TM5 and H216-A229 in TM6. Furthermore, the penetration of water molecules into the receptor core increases the loss of structure in the TM domains by redistribution of H-bonds. The decrease in intramolecular interactions leads to an increase in receptor-water interactions, allowing the opening of the TM domains.

In the same sense, the high fluctuation of the C-terminal domain is promoted by the formation and breaking of H-bonds at the receptor-membrane and receptor-solvent interfaces. The results show that this interaction is stronger in the TIP4P models, whose loading parameters are 18% higher than the three-site models. This increase in activity seen on all water models suggests that the GPR40 could be used as a structural stabilizer or to interact with other biomolecules. However, experimental studies have shown that electrostatic interactions are not dominant near the cell membrane, whose environment is low electrostatic, which could lead to a poor description of the interactions of the receptor with the different surrounding molecular components, impacting its molecular affinity. Remarkably, the flexible FBA/ϵ model does not overestimate this interaction, preserving the helical structures better than the SPC/E model. It should be noted that the FBA/ϵ model has the smallest values in the atomic charges of the four types of water used. These results open the possibility of future work on improving the electrostatic description in these systems.

In our analyses, we seek to relate bulk water properties with the structural and energetic characteristics of the GPR40 receptor. However, receptor-water interactions are complex and can hardly be represented solely by the global dynamics of water as a solvent, in which the hydration layer plays a key role. Even so, computational simulations constitute a powerful tool for its study by offering clues about the mechanisms and interactions that occur at the atomic level. However, the environment surrounding these receptors has not yet been adequately described, and the role that water models play in it has not been sufficiently explored. Without a doubt, more robust studies are necessary; however, exploring new models and the effects they cause on structural properties are essential steps to understanding the dynamics of these receptors.

## Data Availability

Structures and files used in molecular dynamics simulations have been deposited at the following electronic addresses: https://doi.org/10.17632/3tsph9kgsw.1 or https://doi.org/10.17632/3tsph9kgsw.3, with the replicas and pure components information. A list of software used in this study can be found in the Key Resources Table (Table 6)

## Supporting Information Available

Determination of the different domains of the GPR40 structure (Figure S1); plots of the stability indicators obtained from the MD trajectories of the three replicas (RMSD, RMSF, and radius of gyration, Figure S2); stability indicators of the MD simulations of the three replicas (H-bonds, SASA, and MSD, Figure S3); structural and energetic analysis of the GPR40 receptor without considering the C-terminal domain (Figure S4); comparison of the structures obtained at 500 ns of the MD simulations (Figure S5); comparison of the final GPR40 structures with experimental GPCR structures in the active or inactive state (Figure S6); diffusion of the GPR40 receptor and membrane lipid molecules (Figure S7); density profiles of groups that comprise the GPR40-membrane-water complexes (Figure S8); distribution of water molecules in the transmembrane region of the systems (Figure S9); H-bonds between the lipids and the different water models along MD trajectories (Figure S10); plots of H-bonds formation and the dielectric constants of the four water models analyzed (Figure S11). Supplementary Tables: comparison of parameters with other models of GPCRs in inactive or active states (Table S1); top 20 residues with the highest H-bond occupancy in the intramolecular receptor interactions (Table S2); top 20 residues with the highest H-bond occupancy in the GPR40 - solvent interaction (Table S3); and top 20 residues with the highest H-bond occupancy in the GPR40 - lipid interaction (Table S4) (PDF)
