# Supplementary material for: Influence of the Water Model on the Structure and Interactions of the GPR40 Protein with the Lipid Membrane and the Solvent: Rigid versus Flexible Water Models
Source: J Chem Theory Comput. 2024 Jul 11;20(14):6369–87. doi: 10.1021/acs.jctc.4c00571 (PMC11270832; doi:10.1021/acs.jctc.4c00571)
Supplement: Supplementary file 1 — ct4c00571_si_001.pdf [file ct4c00571_si_001.pdf]

Supporting Information

Influence of the water model on the structure and interactions of the GPR40 protein with the lipid membrane and the solvent: rigid versus flexible water models

June 7, 2024

Jorge Alberto Aguilar-Pineda[1‡], and Minerva González-Melchor[1*].
[1] Instituto de Física "Luis Rivera Terrazas", Benemérita Universidad Autónoma de Puebla, Av San Claudio, Edificio IF-1, Ciudad Universitaria, Puebla, Pue. 72570, México; jaguilar@ucsm.edu.pe
Keywords: GPR40 protein; Water models; Molecular Dynamics; Membrane proteins.

*Author to whom correspondence should be addressed.
Electronic mail: minerva@ifuap.buap.mx

Supporting Figures

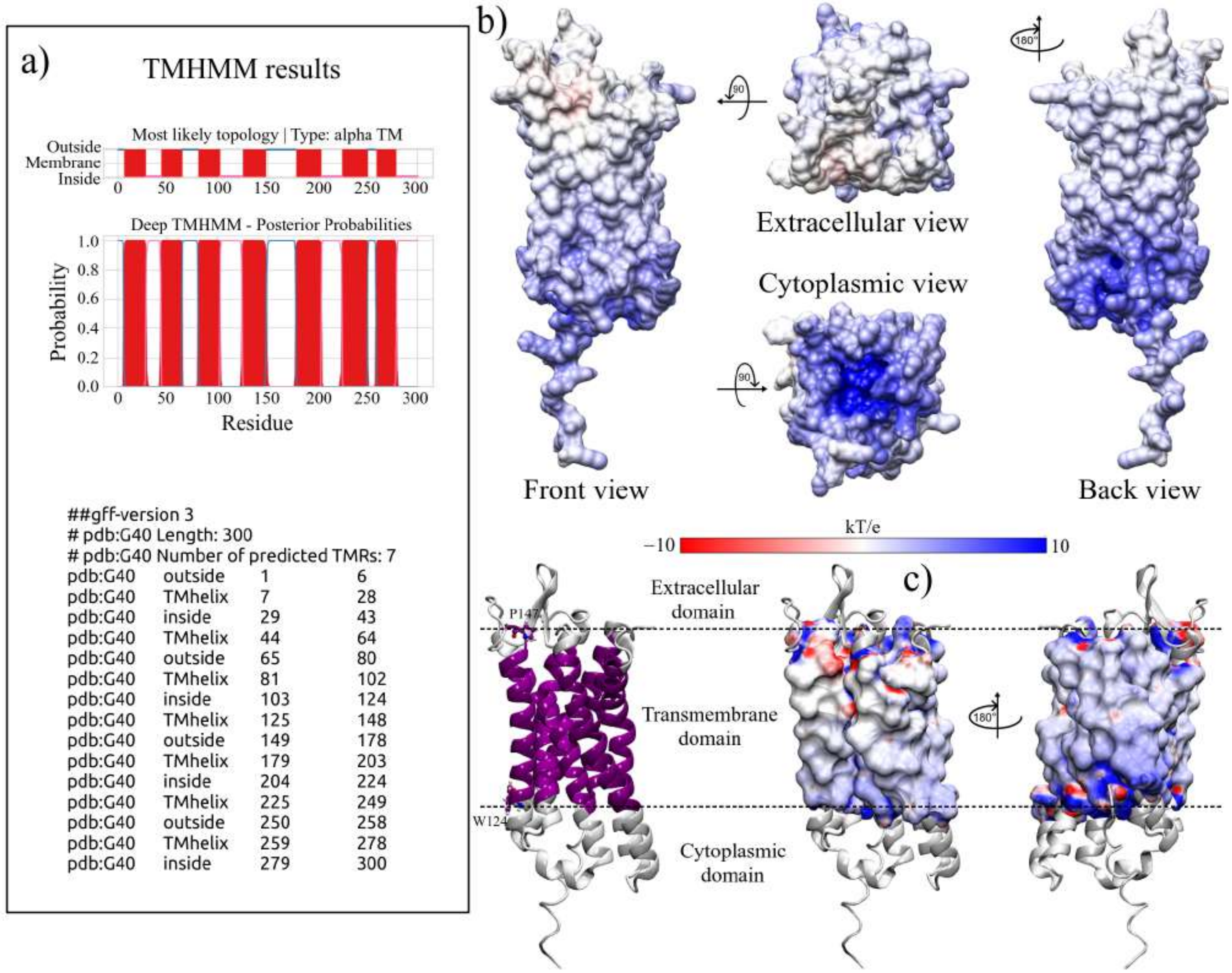

Figure S1. Determination of the different domains of the GPR40 structure. a) Results obtained from the DeepTMHMM server. The graphs show the probability that a residue is located in the extracellular (Outside, blue), transmembrane (Membrane, red), or intracellular or cytoplasmic (Inside, pink) region. In addition, the server indicates the residues that make up each domain. b) Electrostatic potential mapped on the surface of the protein. The colors indicate electrophilic (blue), hydrophobic (white), and nucleophilic (red) zones. The distance between residues W124 and P147 determined membrane thickness.

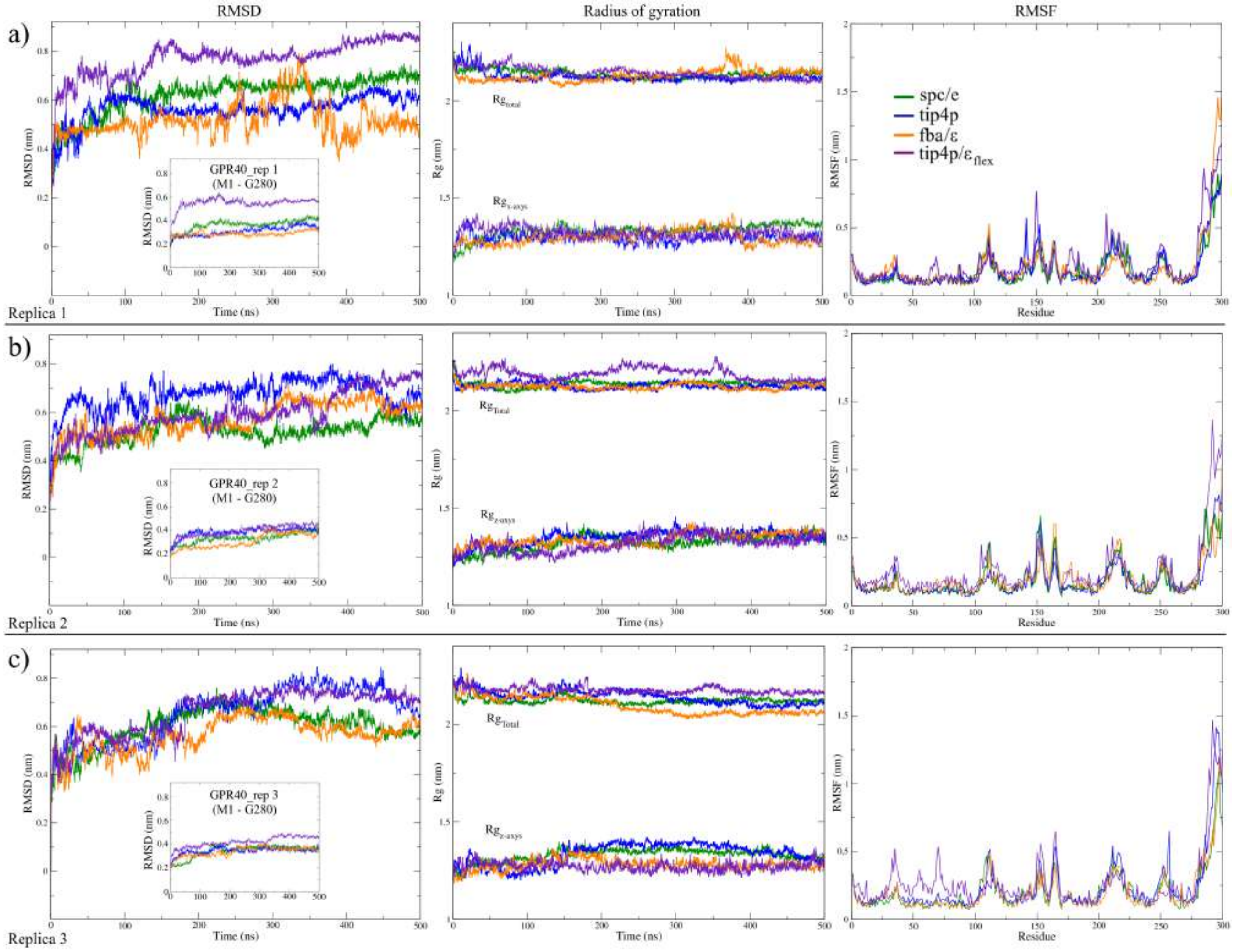

Figure S2. Plots of the stability indicators obtained from the MD trajectories of the three replicas analyzed in this work. The plots shown are of RMSD (left panel), the radius of gyration (middle panel), and RMSF (right panel). The RMSD insets show the values of this indicator without considering the residues of the C-terminal domain; that is, the calculated structure includes residues Met1 to Gly280. To compare, in both plots, the same scale was used for the x and y axes.

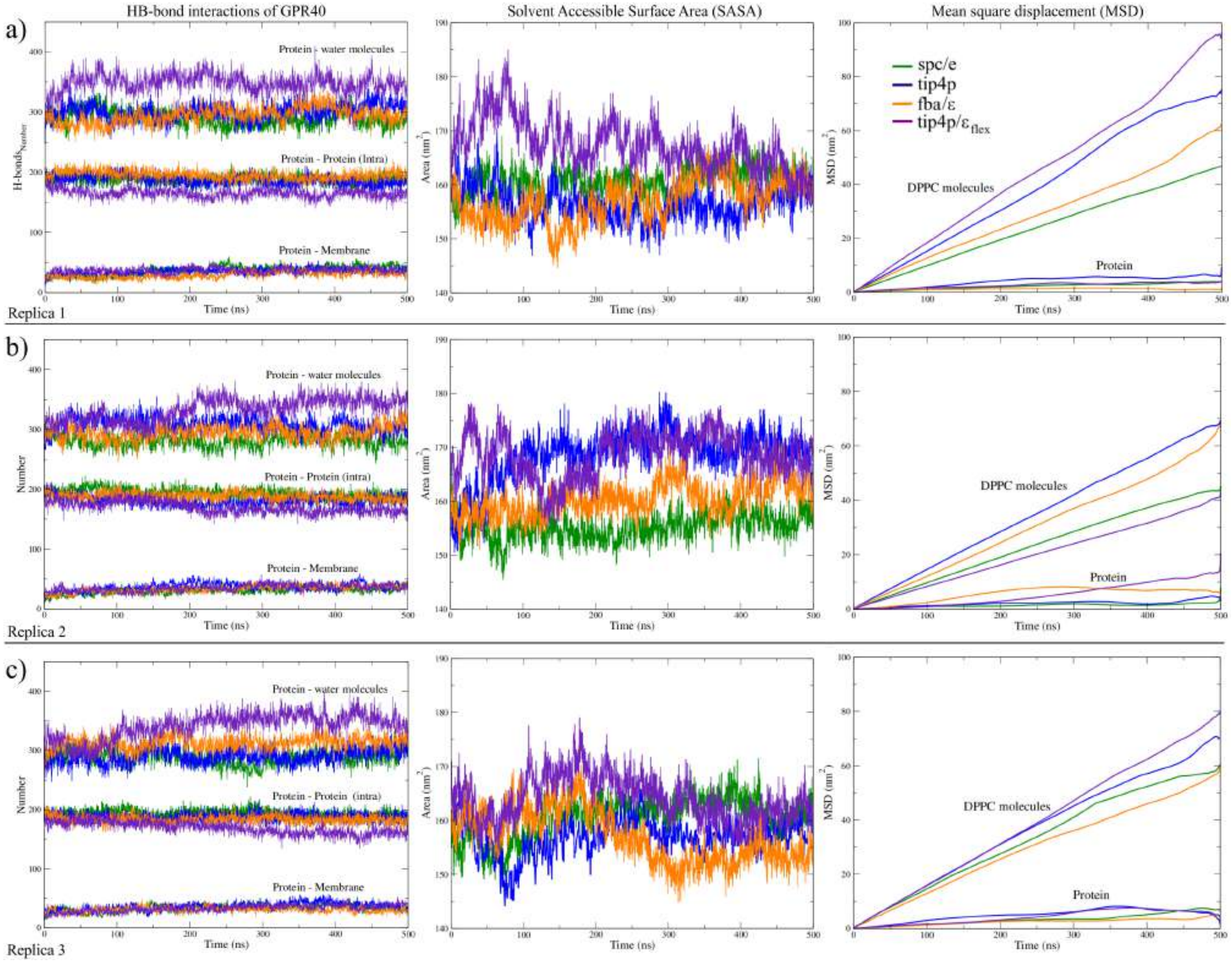

Figure S3. Stability indicators of the MD simulations of the three replicas carried out in the present work. The plots shown are of the H-bonds (left panel), SASA (middle panel), and MSD (right panel).

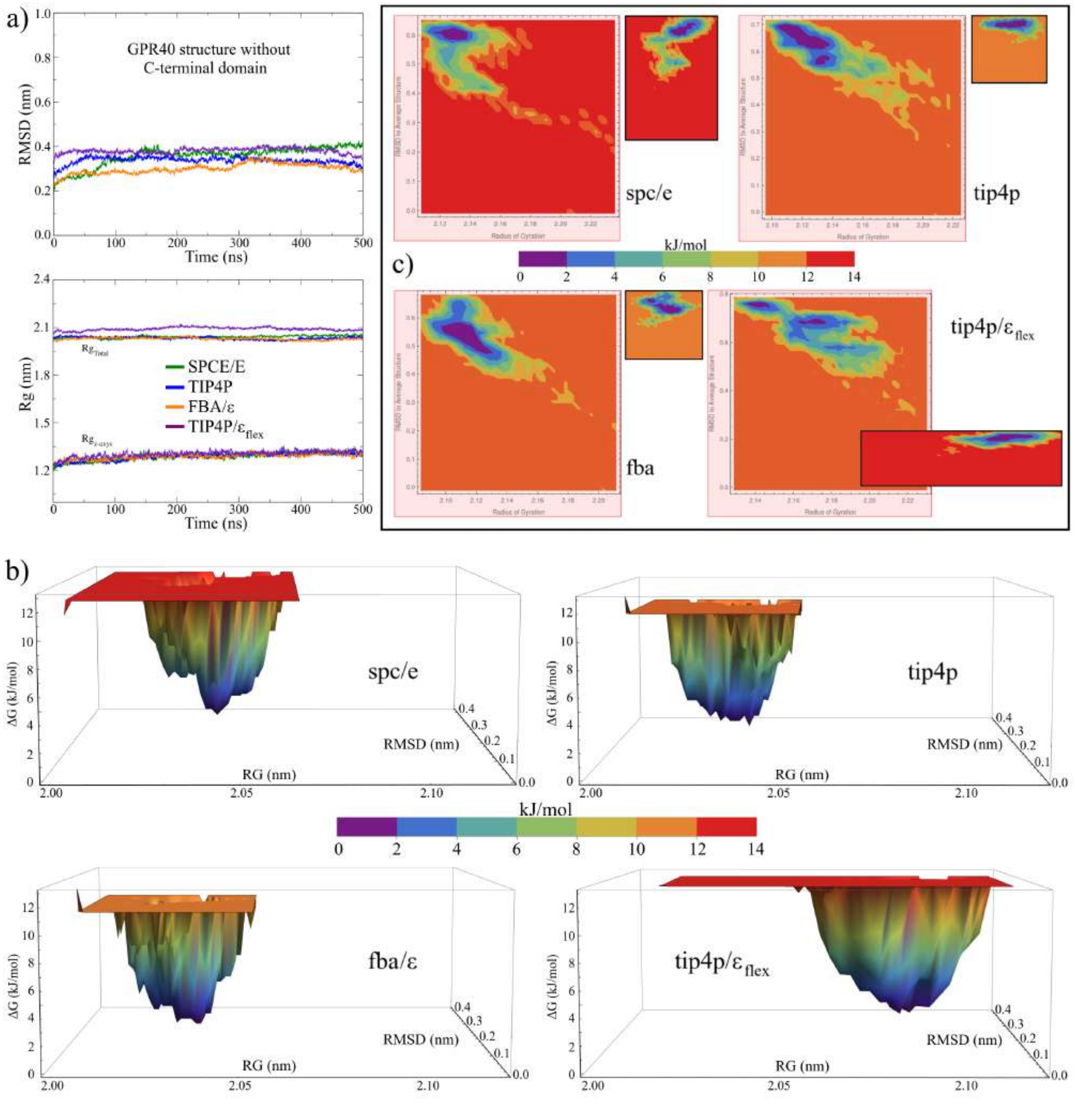

Figure S4. Structural and energetic analysis of the GPR40 receptor without considering the C-terminal domain. a) Plots of root mean square deviation ($RMSD_{avg.}$) and radius of gyration ($RG_{avg.}$) averages obtained from the MD simulations with 500 ns trajectories. The color of the lines indicates the water model used in the systems. b) Analysis of the free energy landscape (FEL), taking the $RMSD_{avg.}$ and $RG_{avg.}$ calculations as structural variables. The axes show the same scales of the three calculated variables for better comparison. c) Comparison of the different surfaces obtained in the FEL analysis. The areas on the left side of each model correspond to the complete structure of the receiver. Those on the right side, and with a smaller surface area, to the receptor without the C-terminal domain. In both cases, the energy values scale and range of colors were preserved.

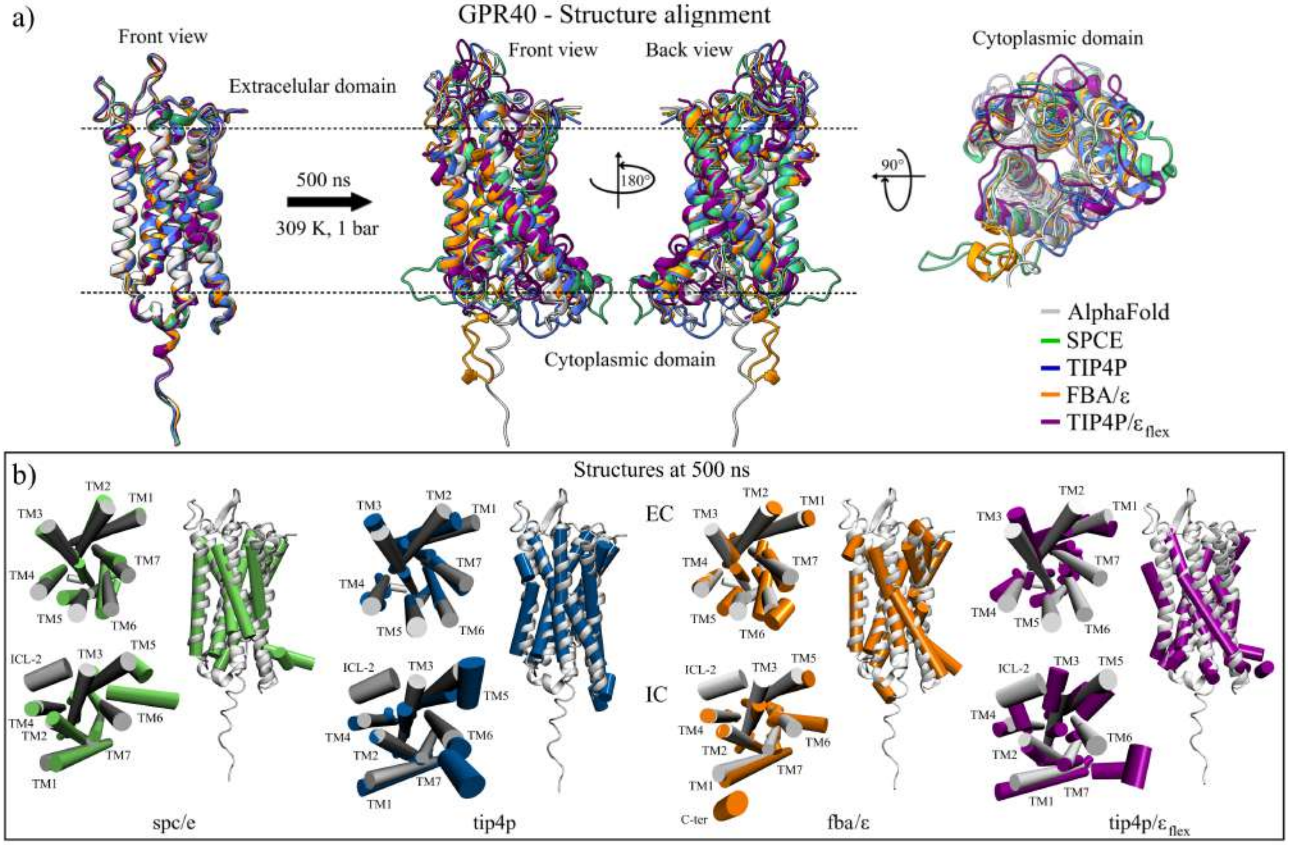

Figure S5. Comparison of the structures obtained at 500 ns of the MD simulations. a) Structural alignment of the GPR40 proteins obtained at the beginning and end of the MD trajectories. The AlphaFold model structure was taken as the basis for the RMSD calculations of each configuration. b) Analysis of the conformational changes of each model studied. The alignment shows the opening of the final structures to take a configuration active-like state.

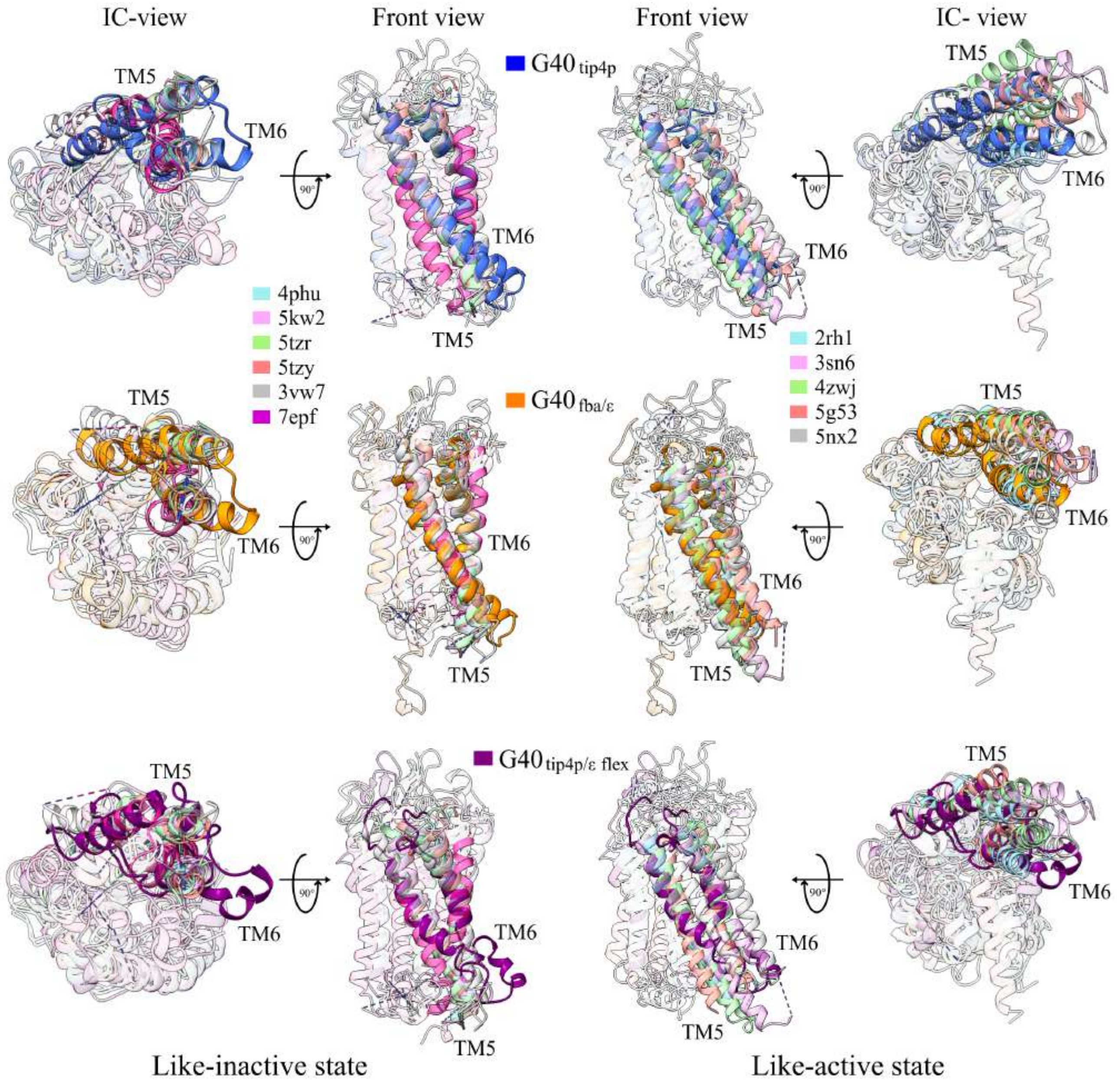

Figure S6. Comparison of the final GPR40 structures with experimental GPCR structures in the active or inactive state. The alignments correspond to the TIP4P (top panel), FBA/ϵ (middle panel), and TIP4P/ϵ_{flex} (bottom panel). The colors of the GPR40 are the same as those used previously.

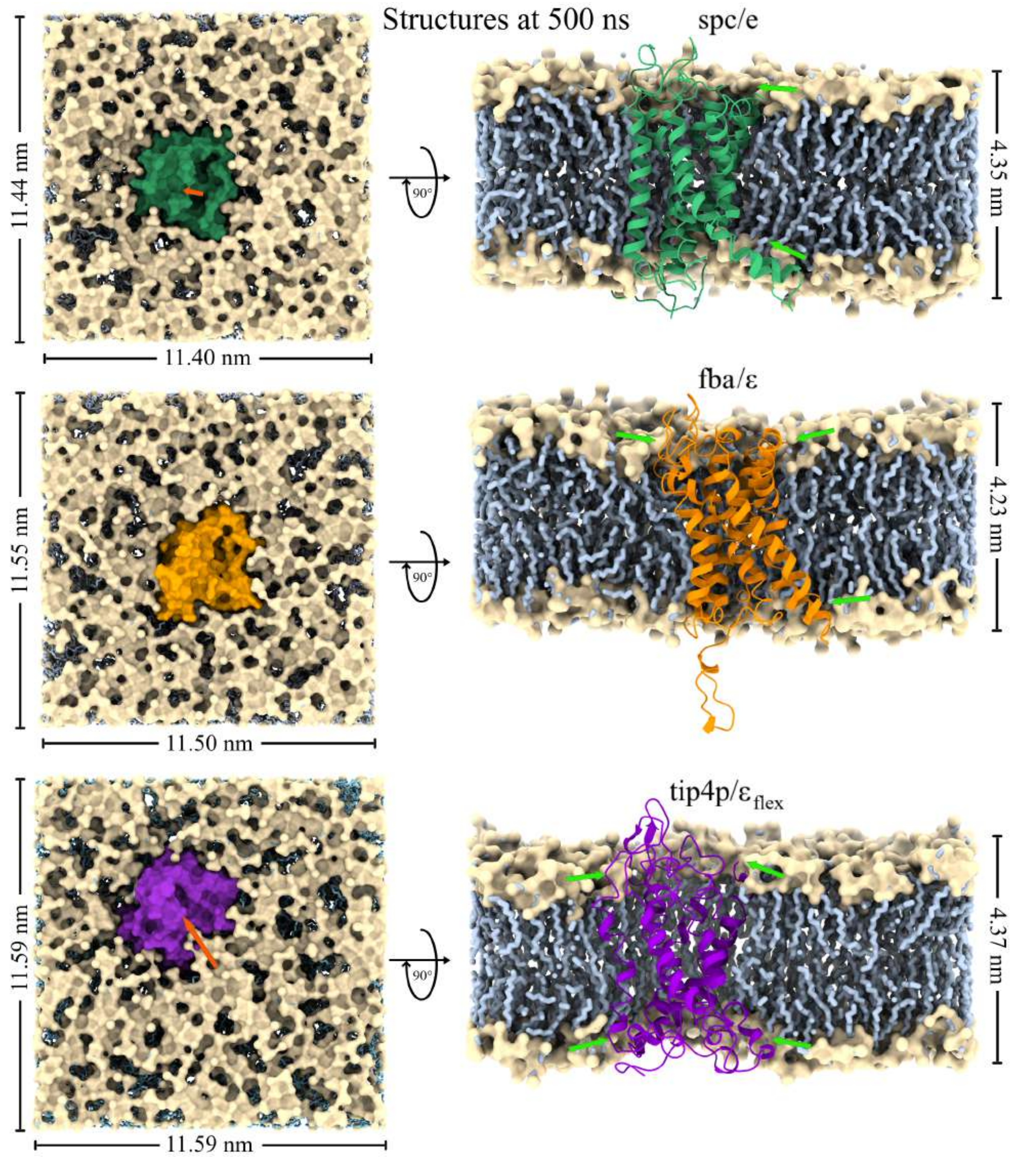

Figure S7. Diffusion of the GPR40 receptor and membrane lipid molecules. The figures in the left panel show the lateral diffusion along the z-axis of the receptor. The orange arrows indicate the main direction of translational motion. The final dimensions of the membrane due to its compaction are also shown. The figures in the right panel show the movement of lipids towards the receptor structure (green arrows) and their compaction.

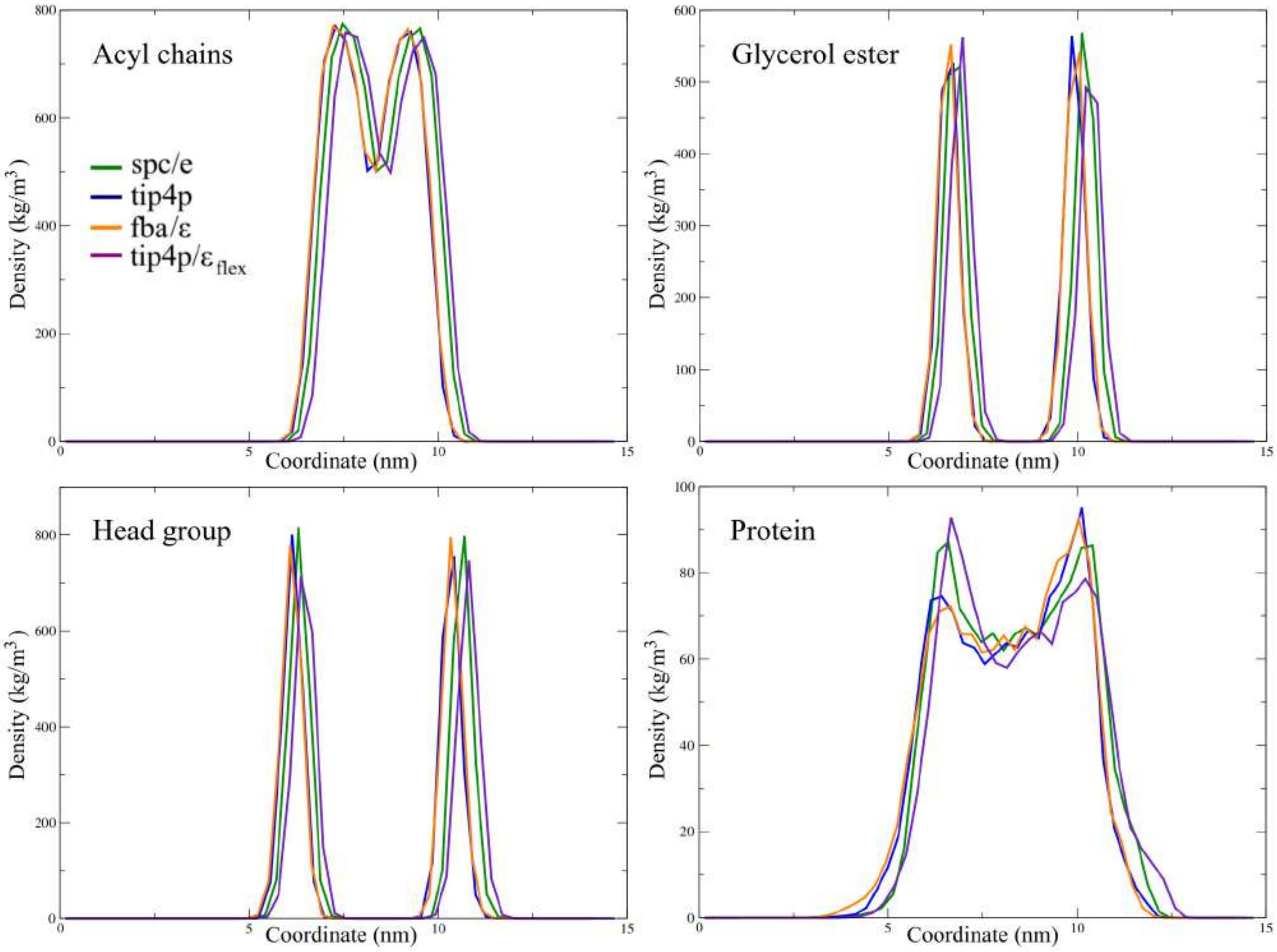

Figure S8. Density profiles of groups that comprise the GPR40-membrane complexes.

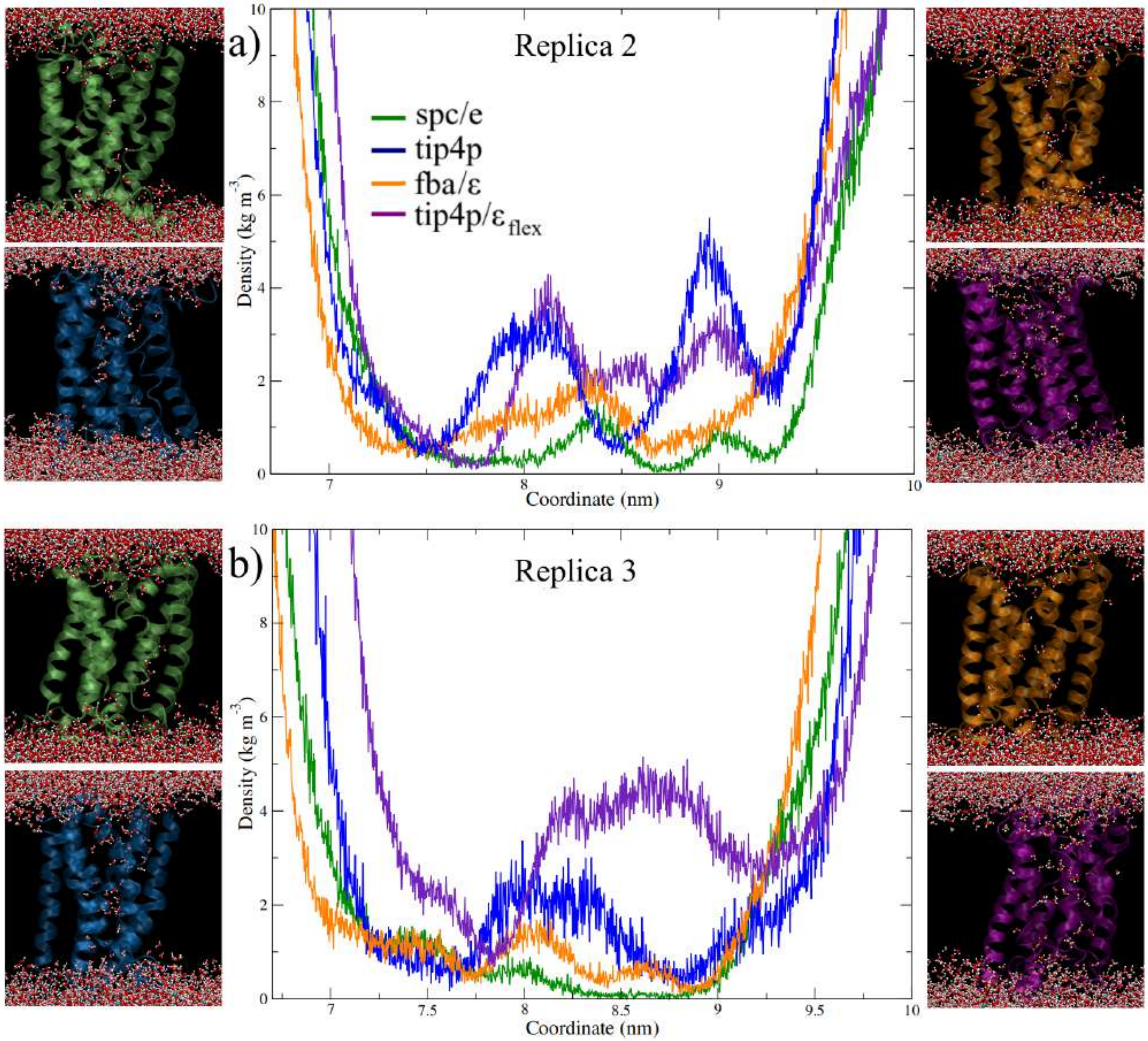

Figure S9. Distribution of water molecules in the transmembrane region of the systems. a) Replica 1. b) Replica 2. The images correspond to the last frame of the MD trajectories (500 ns).

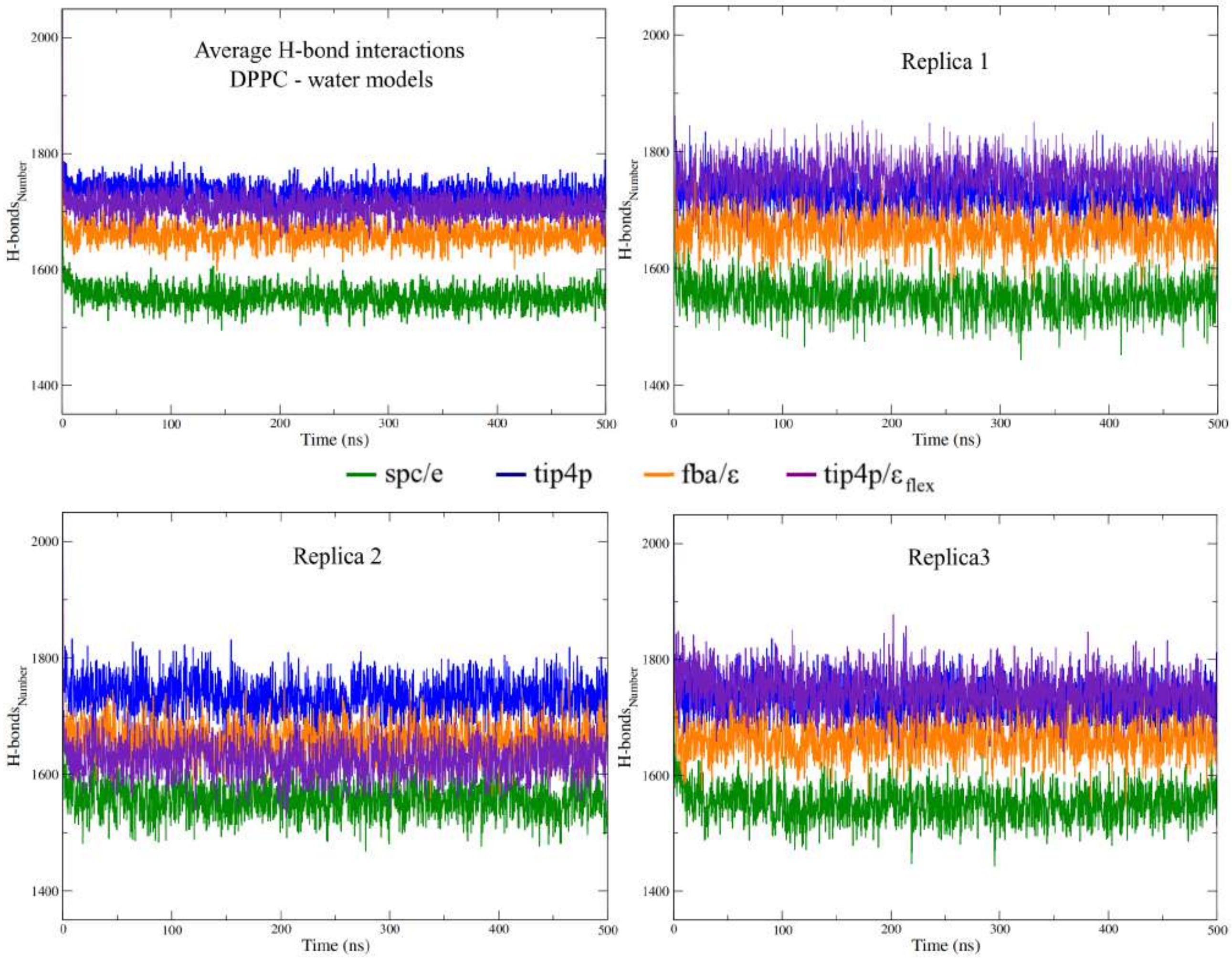

Figure S10. Hydrogen bonds between the lipids and the different water models along MD trajectories.

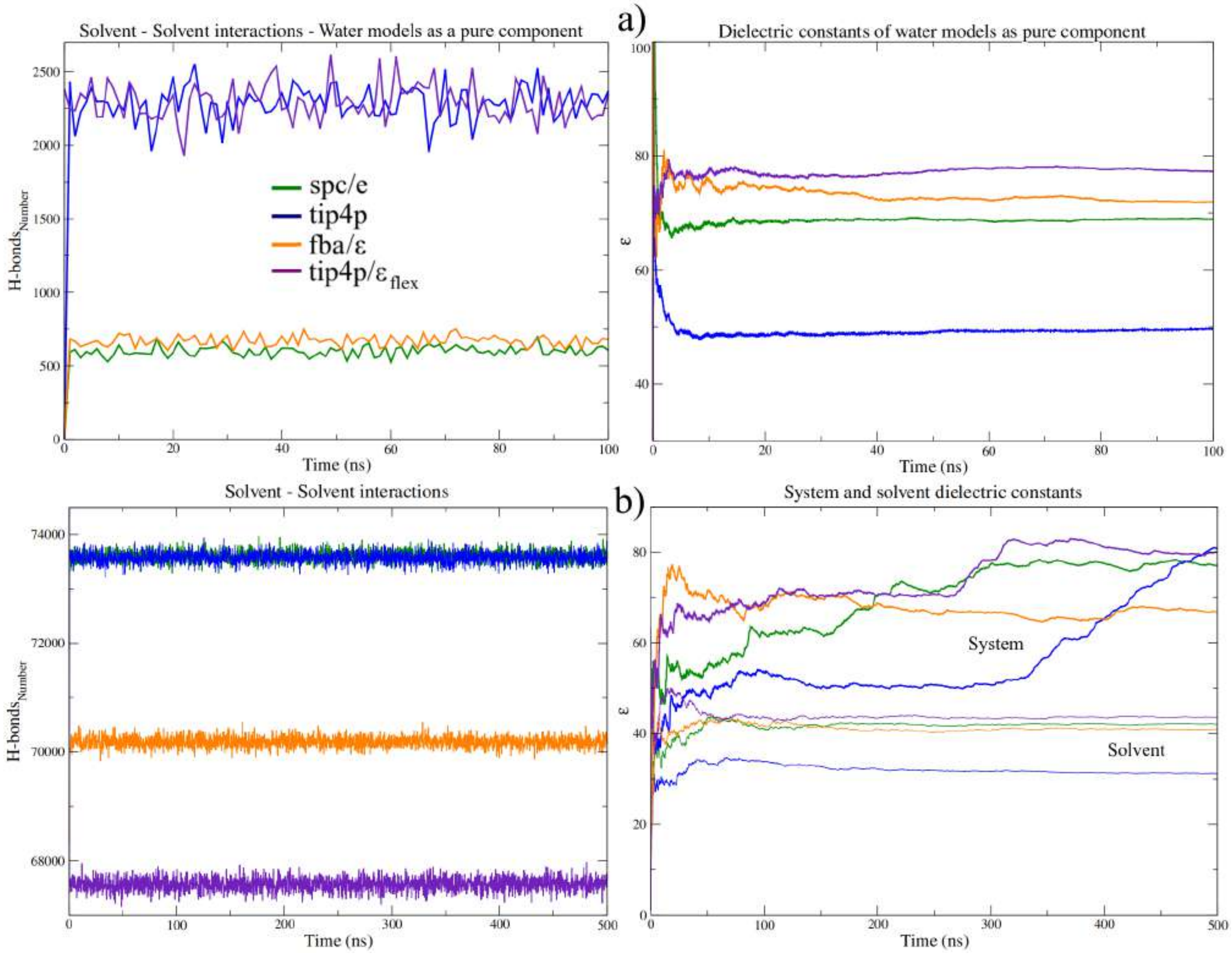

Figure S11. Graphs of H-bonds formation and the dielectric constants of the four water models analyzed. a) Pure component. Calculations were performed with 500 water molecules from each model with MD trajectories of only 100 ns. b) Systems studied. The curves were obtained considering all the system components (System, thick lines) and choosing only the water molecules (Solvent, thin lines). The simulation temperature was 309.65 K at 1 pressure bar in both cases.

Supporting Tables

Table S1. Comparison of parameters with other models of GPCRs in inactive or active states.

Model	Inactive-like state					Active-like state			
	5kw2	4phu	5tzr	5tzy	Average	8ejk	8eit	8ejc	Average
SPC/E	3.833	3.863	4.025	3.833	**3.889**	2.951	3.212	3.036	**3.066**
TIP4P	3.636	2.978	3.046	3.379	**3.260**	2.837	3.067	2.980	**2.961**
FBA/ϵ	3.394	2.805	2.940	3.107	**3.062**	2.665	2.834	2.773	**2.757**
TIP4P/ϵ_{flex}	5.620	5.214	5.198	5.583	**5.404**	4.629	4.778	4.819	**4.742**

Table S2. Top 20 residues with the highest H-bond occupancy in the intramolecular receptor interactions

	SPC/E		TIP4P		FBA/ϵ		TIP4Pϵ_{flex}	
	Residue	% Occ.	Residue	% Occ.	Residue	% Occ.	Residue	% Occ.
1	R258	595.6	R258	550.5	I27	433.1	N23	454.3
2	E172	514.2	E172	523.4	R258	430.4	H33	438.7
3	N272	466.7	N23	502.2	S185	405.6	R183	399.6
4	S185	439.9	N272	447.9	S187	366.1	N272	367.3
5	N23	415.2	I27	403.2	E172	361.3	H137	304.2
6	I27	408.0	H33	397.6	N23	354.9	S268	304.2
7	H33	397.2	S185	388.8	T31	354.1	R37	297.8
8	D175	380.8	D175	359.7	S268	349.7	G19	291.8
9	D152	328.6	S187	355.7	N272	341.7	F16	291.0
10	T31	322.2	E65	325.4	D175	337.7	I27	290.6
11	S8	304.6	T31	308.6	H33	321.4	R258	287.8
12	S187	301.8	S268	300.6	A92	320.2	N161	279.1
13	L260	298.2	R183	297.8	S8	313.4	D175	278.7
14	T276	295.0	K62	295.0	F20	299.0	L106	273.9
15	E65	289.0	C201	294.2	E65	295.4	G265	271.5
16	F20	281.8	F9	280.6	T198	291.0	T31	269.1
17	A92	281.1	F20	274.7	F9	290.2	Q115	265.5
18	V13	281.0	V24	274.7	R183	287.9	K300	265.5
19	F9	271.5	S8	273.1	Y202	281.4	R104	262.7
20	V24	267.5	T198	270.7	L260	280.3	F192	256.3

Table S3. Top 20 residues with the highest H-bond occupancy in the GPR40 - Solvent interaction.

	SPC/E		TIP4P		FBA/ϵ		TIP4Pϵ_{flex}	
	Residue	% Occ.	Residue	% Occ.	Residue	% Occ.	Residue	% Occ.
1	K300	881.2	D152	1439.0	K300	818.8	D152	1514.6
2	E145	601.0	K300	1437.0	E145	745.9	E145	1495.1
3	D2	594.4	E145	1417.7	D2	637.8	D2	1471.8
4	E172	550.9	D2	1307.8	E172	577.4	E172	1324.7
5	D175	518.6	E172	1216.6	D175	539.8	K300	1145.0
6	R104	480.4	D175	1140.4	D152	458.6	E65	963.0
7	E65	437.4	E65	925.0	R292	430.8	D175	888.3
8	R37	425.4	R292	878.8	E65	426.0	D52	806.4
9	R292	414.4	N165	770.8	R118	408.0	N165	752.0
10	R118	406.0	R37	758.0	N165	407.0	W150	740.6
11	R218	405.4	R118	729.6	R218	399.4	R218	704.8
12	R183	401.8	R211	717.2	R211	386.0	R211	693.4
13	R211	391.8	Q299	711.6	R104	377.3	R207	688.0
14	H153	386.2	R183	692.0	R281	371.4	K286	658.2
15	R221	361.2	N252	638.6	R37	354.4	R292	650.0
16	D152	356.4	R104	629.2	R183	348.1	Q299	638.0
17	N165	345.6	N155	612.8	Q294	338.4	Q294	635.6
18	R281	343.0	R218	602.9	R119	323.6	R104	632.8
19	K286	342.0	H153	590.8	H153	321.2	S298	621.6
20	R119	309.6	S167	562.6	N155	319.8	R281	612.8

Table S4. Top 20 residues with the highest H-bond occupancy in the GPR40 - Lipid interaction.

	SPC/E		TIP4P		FBA/ϵ		TIP4Pϵ_{flex}	
	Residue	% Occ.	Residue	% Occ.	Residue	% Occ.	Residue	% Occ.
1	R217	193.3	R281	312.0	R207	206.4	R35	239.6
2	R281	191.2	R217	311.2	R217	170.7	R217	220.6
3	R35	187.0	R35	253.5	R28	151.3	R119	188.1
4	R207	172.1	R28	185.3	R35	134.0	R28	188.0
5	R28	136.3	R207	163.7	R221	99.2	Q115	143.5
6	H216	102.2	R211	125.2	R281	90.5	R221	114.6
7	Q6	93.6	M1	115.6	R119	82.7	M1	105.2
8	Q294	93.4	R119	104.4	K219	81.4	H216	98.6
9	S77	92.8	S77	79.5	R211	80.3	R207	93.2
10	R211	89.0	K219	78.6	H216	79.9	Q6	90.9
11	R221	85.4	S256	78.2	Q6	78.3	S77	89.9
12	G113	81.2	Q6	75.3	S77	69.1	R211	88.9
13	F117	77.3	C121	72.5	M1	67.5	Y114	66.5
14	G254	76.5	Y122	67.5	Y122	66.3	S157	65.9
15	R119	69.1	T287	66.3	C121	62.9	R281	64.1
16	A116	68.1	H216	65.7	Y105	62.7	G254	62.7
17	C121	67.3	Q294	54.7	L38	59.7	W257	60.7
18	R292	66.3	Y250	54.5	A116	58.1	A182	58.3
19	Y122	65.1	G255	54.3	W150	54.7	G280	54.7
20	K219	63.9	R221	53.3	N252	52.7	D2	52.1
